# Monkeypox Knowledge and Vaccine Hesitancy of Czech Healthcare Workers: A Health Belief Model (HBM)-Based Study

**DOI:** 10.3390/vaccines10122022

**Published:** 2022-11-26

**Authors:** Abanoub Riad, Anton Drobov, Jana Rozmarinová, Pavla Drapáčová, Jitka Klugarová, Ladislav Dušek, Andrea Pokorná, Miloslav Klugar

**Affiliations:** 1 Czech National Centre for Evidence-Based Healthcare and Knowledge Translation (Cochrane Czech Republic, Czech EBHC: JBI Centre of Excellence, Masaryk University GRADE Centre), Faculty of Medicine, Masaryk University, 625 00 Brno, Czech Republic; 2 Institute of Health Information and Statistics of the Czech Republic (IHIS-CR), 128 01 Prague, Czech Republic; 3 Department of Public Health, Faculty of Medicine, Masaryk University, 625 00 Brno, Czech Republic; 4 Department of Health Sciences, Faculty of Medicine, Masaryk University, 625 00 Brno, Czech Republic

**Keywords:** cross-sectional studies, Czech Republic, disease outbreaks, health belief model, health personnel, knowledge, monkeypox, smallpox vaccine, vaccination hesitancy

## Abstract

Background: The recent human monkeypox virus (HMPXV) outbreak in non-endemic countries that started in May 2022 has raised concerns among public health authorities worldwide. Healthcare workers (HCWs) play a decisive role during epidemics in transmitting accurate information to the public and motivating them to pursue protective behaviours, including immunisation. Methods: A cross-sectional survey-based study was conducted in the Czech Republic in September 2022 to evaluate HMPXV-related knowledge and vaccination perceptions among HCWs. The study utilised a digital self-administered questionnaire (SAQ) to collect data from the target population. The proposed SAQ inquired about participants’ sociodemographic and anamnestic characteristics, perceived knowledge of HMPXV, factual knowledge, and vaccination perceptions according to the health belief model (HBM). Results: A total of 341 participants were included in this study; most of them were females (88.9%), allied HCWs (89.4%), heterosexuals (87.1%), married (61.9%), and vaccinated against COVID-19 (91.2%). Only 8.8% of the participants agreed to receive vaccination against HMPXV; 44.9% rejected it, while 46.3% were hesitant. While digital news portals (47.5%) and social media (25.8%) were among the most utilised sources of information about HMPXV, the scientific journals (5.6%), ECDC (5%), and the U.S. CDC (1.5%) were the least common sources. The participants demonstrated suboptimal levels of factual knowledge, especially regarding HMPXV vaccines (1.5 ± 1.2 (0–4)) and treatments (0.9 ± 0.9 (0–4)). Additionally, several misconceptions were detectable among the participants, regarding topics such as the availability of effective vaccines and antivirals against HMPXV, the risk of vertical transmission, and homosexual stigmatisation. The HBM indicated that the cues to action and perceived susceptibility were the most important constructs to predict HMPXV vaccine acceptance. Conclusions: the findings of this study call upon public health practitioners and health policymakers in the Czech Republic to act accordingly in order to determine the drivers of vaccine hesitancy among Czech HCWs. Dedicated educational campaigns should aim to counter the HCWs’ misconceptions around HMPXV, and future studies should aim to explore the prevalence and drivers of HMPXV vaccine hesitancy among the general population.

## 1. Introduction

While the world is still challenged by the coronavirus disease (COVID-19) pandemic, the emergence of a new outbreak caused by the human monkeypox virus (HMPXV) has raised global concerns among public health authorities [1]. HMPXV is a viral zoonosis with clinically less severe symptoms than previously reported in patients with smallpox [2]. As smallpox was eradicated in 1980 and the vaccination against this disease has subsequently ceased, HMPXV has emerged as the most important *orthopoxvirus* for public health [2]. HMPXV is a disease of global public health importance, as it affects not only countries in West and Central Africa—where it is endemic—but also the rest of the world [2]. Since May 2022, there have been multiple cases of HMPXV identified in non-endemic countries, which has led to an intensive investigation at both international and national levels for a better understanding of its infection sources and transmission patterns [2,3]. Subsequently, the World Health Organization (WHO) declared the global HMPXV outbreak as a public health emergency of international concern on 23 July 2022 [4]. As of 21 November 2022, 80,328 laboratory-confirmed cases of HMPXV have been identified worldwide, including 70 cases in the Czech Republic [5].

Healthcare workers (HCWs) are considered to be a high-risk group for infectious disease transmission by the centres of disease control in the European Union (EU) and the United States (US) [2,6]. In order to stop human-to-human transmission and to minimise zoonotic transmission of HMPXV, vaccination can be used as a primary method of prevention [4]. According to the WHO’s interim guidance on vaccines and immunisation for HMPXV, updated on 24 August 2022, mass vaccination was not recommended nor required for HMPXV at that moment [4]. Meanwhile, the WHO recommended that pre-exposure prophylaxis (PrEP) should be administered to high-risk groups, including (a) HCWs at high risk of exposure, (b) laboratory personnel working with orthopoxviruses, (c) clinical laboratory personnel performing diagnostic testing for HMPXV, and (d) outbreak response team members (as designated by national public health authorities) [4]. Thus, the risk could be perceived as higher in bedside allied HCWs helping patients with basic daily activities (e.g., washing patients and making beds).

A high-risk exposure can be defined as “direct exposure of the skin or mucous membranes to the skin or respiratory secretions of a person with HMPXV, their body fluids or potentially infectious material including clothing and bedding, without appropriate personal protective equipment (PPE)”. Therefore, high-risk exposure is predictable within clinical settings through various sources such as inhalation of droplets, mucosal exposure to splashes, and penetrating sharp injuries. The spread of HMPXV from person to person has been known to generally require prolonged close contact, such as face-to-face contact in close proximity or skin-to-skin physical contact. Such exposure can easily occur in healthcare settings, which puts HCWs at risk of contracting the disease [4].

Some endemic countries for HMPXV (such as the Democratic Republic of the Congo) have launched routine HMPXV surveillance, where one of the main parts of this procedure is to enhance the capacity of HCWs to identify cases and improve patient management [7]. Hence, HCWs should have sufficient knowledge of HMPXV in order to be able to immediately identify, report, and manage new cases to prevent further transmission. Considering that the Czech Republic has not been endemic for HMPXV, there might be a lack of knowledge on this particular disease among HCWs, since it has not been deeply studied and explained in educational institutions. As in Indonesia, general practitioners had low knowledge of HMPXV in 2020 [8]. In addition, it was mentioned in the WHO’s report that one of the challenges faced in preventing the re-emergence of HMPXV was a lack of knowledge—particularly among HCWs [9]. Since cases of HMPXV have already been registered in the Czech Republic, it is crucial for HCWs to be informed, knowledgeable, and prepared for HMPXV case management. HCWs play an instrumental role in primary prevention and health promotion; therefore, their knowledge about emerging infectious diseases may influence their perceptions and attitudes towards their patients during these critical times [10]. In addition, vaccine hesitancy among HCWs can not only slow down the public health strategies that aim to protect high-risk groups and individuals, but it may also undermine public confidence in vaccines for orthopoxviruses.

The overarching goal of this study was to evaluate levels of HMPXV-related knowledge and HMPXV vaccine perceptions among Czech HCWs. The primary objectives included (a) to assess the levels of perceived knowledge and factual knowledge about HMPXV, (b) to evaluate perceptions of vaccination against HMPXV according to the health belief model (HBM), and (c) to assess levels of HMPXV vaccine acceptance and willingness to pay for it. The secondary objectives were (a) to explore the potential sociodemographic and anamnestic predictors of HMPXV-related knowledge and HMPXV perceptions, and (b) to discover the knowledge gaps among Czech HCWs in terms of HMPXV.

## 2. Materials and Methods

### 2.1. Design

The present study was designed as an analytical cross-sectional study executed and reported according to the Strengthening the Reporting of Observational Studies in Epidemiology (STROBE) guidelines [11].

### 2.2. Settings

A survey-based study was carried out in September 2022, utilising a self-administered questionnaire (SAQ) to collect data from Czech HCWs about their human monkeypox virus (HMPXV)-related knowledge and vaccination perceptions. The SAQ was coded and disseminated online through KoBoToolbox (Harvard Humanitarian Initiative; Cambridge, MA, USA, 2022) [12].

### 2.3. Participants

The target population of the present study were Czech HCWs who may/may not provide clinical care to HMPXV cases during the 2022 outbreak.

The inclusion criteria were (i) to be a full-time or part-time employee at a healthcare provider in the Czech territories, and (ii) to be responsible for providing clinical services. The exclusion criteria were (i) to be administrative, economic, or legal staff working at Czech healthcare providers; (ii) to be research personnel uninvolved in providing clinical care; and (iii) to be an undergraduate healthcare student.

A non-random sampling strategy utilising the snowballing technique was used to recruit respondents from the target population. Official email invitations were sent to the heads of Czech medical societies that are members of the Czech Medical Association of J. E. Purkyně (CzMA), as well as the managers of inpatient healthcare facilities within the network of the Central Adverse Events Reporting System of the Institute of Health Information and Statistics of the Czech Republic (IHIS-CR; Prague, Czech Republic), in order to facilitate participation in the study by circulating the survey’s uniform resource locator (URL) through their respective networks. In addition, online advertisements and blog posts were published by the official websites of the Czech Ministry of Health (MoH) and the Faculty of Medicine, Masaryk University (MED-MUNI) [13,14].

The minimum sample size required for this study was estimated using Epi Info^TM^ version 7.2.5 (CDC, Atlanta, GA, USA, 2021) utilising the following assumptions: (i) confidence level (CI): 95%; (ii) acceptable error margin: 5%; (iii) target population size: >250,000 [15], and (iv) expected frequency of the primary outcome (i.e., HMPXV vaccine acceptance): 70% [16].

At least 322 valid responses were required to establish an inference between putative demographic and anamnestic predictors and the current intentions of Czech HCWs to receive an HMPXV vaccine. A total of 344 responses were received, out of which only 3 were excluded because of a lack of consent and/or information (Figure S1).

### 2.4. Instrument

The SAQ of this study consisted of 55 closed-ended items divided into eight categories:

(i) Sociodemographic characteristics: gender, sexual orientation, age, profession, marital status, having minors (≤18 years old), and providing care to HMPXV patients.
(ii) Anamnestic characteristics: chronic illnesses, regular medications, COVID-19 vaccination, and seasonal influenza vaccination.
(iii) HMPXV information sources: undergraduate education, information sources and their confidence levels.
(iv) HMPXV perceived knowledge: HMPXV epidemiology, clinical presentation, risk factors, vaccination, and treatment.
(v) HMPXV factual knowledge: HMPXV epidemiology, clinical presentation, risk factors, vaccination, and treatment.
(vi) HMPXV vaccine perceptions according to the health belief model (HBM): perceived susceptibility, perceived severity, perceived benefits, perceived barriers, and cues to action.
(vii) HMPXV vaccine intentions: acceptance and recommendation to others.
(viii) Willingness to pay (WTP) for HMPXV vaccine.

The draft SAQ was developed according to published studies on HMPXV and HMPXV vaccines among HCWs and other population groups [17,18,19,20]. The content validity of the draft SAQ had been evaluated by a committee of experts in public health, infectious diseases, and health psychology, who provided feedback and suggested modifications for the final version. The construct’s validity was checked by confirmatory factor analysis (CFA), which suggested a good fit of the model (TLI: 0.904; RMSEA: 0.046—CI 95%: 0.040–0.051).

### 2.5. Measures

The level of confidence in sources of HMPXV information was evaluated by a 7-point Likert scale ranging from (extremely unreliable = 1) to (extremely reliable = 7). Similarly, the HMPXV perceived knowledge items, the HBM items, and the HMPXV vaccine intentions were rated using 5-point Likert scales ranging from (strongly disagree = 1) to (strongly agree = 5).

A total of 15 multiple-choice questions (MCQs) were used to evaluate the HMPXV factual knowledge of the participants—3 items for each knowledge domain. Out of these 15 MCQs, 10 items had a single correct answer, which gave them a binary rating (true = 1; false = 0). Five MCQs had more than one correct answer; therefore, their rating had three levels (advanced knowledge = 2; acceptable knowledge = 1; no knowledge = 0).

### 2.6. Ethics

The Ethical Committee of the Faculty of Medicine, Masaryk University, reviewed and approved the protocol of this study on 19 September 2022, with the reference number 73/2022. The Declaration of Helsinki and the European Union (EU)’s General Data Protection Regulation (GDPR) were followed during data collection and processing [21,22].

### 2.7. Analyses

The categorical variables—such as gender, sexual orientation, and profession—were reported using frequencies (*n*) and percentages (*%*), while ordinal and numerical variables—such as confidence levels, perceptions, and knowledge scores—were reported using means and standard deviations (*µ* ± *SD*). The normal distribution of numerical and ordinal variables was tested using the Shapiro–Wilk test with a significance level (*p*) ≤ 0.05. Inferential statistics were performed using the chi-squared (*χ*^2^) test, Fisher’s exact test, analysis of variance (ANOVA), the Kruskal–Wallis (*H*) test, and the Mann–Whitney (*U*) test with *p* ≤ 0.05. The model fit of the HBM was evaluated using structural equation modelling (SEM). All statistical analyses were executed through the Statistical Package for Social Sciences (SPSS) version 28.0 (SPSS Inc. Chicago, IL, USA, 2020) and the R-based open software jamovi [23,24].

## 3. Results

### 3.1. Demographic Characteristics

A total of 341 participants were included in this study, out of which females were the majority (88.9%), followed by males (9.7%) and participants who rejected to disclose their gender identity (1.5%). Regarding the sexual orientation of the participants, the majority were heterosexuals (87.1%), followed by homosexuals (2.3%), bisexuals (1.5%), and non-disclosing participants (9.1%). The mean age of the study sample was 46.1 ± 12.0 years old, and the most common marital statuses were being married (61.9%), single (19.1%), and divorced (12%).

While most participants were allied HCWs (89.4%), only 4.7% reported that they were either currently or potentially providing clinical care to monkeypox patients. More than 37% of the participants reported having minors (below 18 years old) (Table 1).

**Figure 1:**
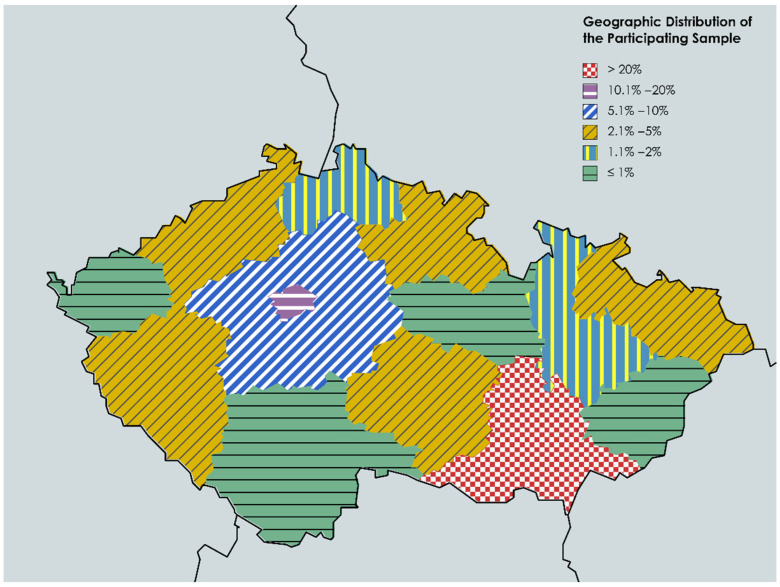
Geographic distribution of Czech HCWs participating in the HMPXV survey, September 2022 (*n* = 341).

The South Moravian Region was the most represented geographical region (59.2%), followed by the capital city Prague (14.1%), the Central Bohemian Region (8.5%), and the Moravian–Silesian Region (3.5%). There was no statistically significant difference between South Moravian Region respondents and other regions’ respondents in terms of HMPXV vaccine acceptance (*p* = 0.076) (Figure 1).

### 3.2. Anamnestic Characteristics

When asked about their medical anamnesis, 38.7% and 47.2% of the participants reported suffering from at least one chronic illness and receiving at least one regular medication, respectively. Among the 132 participants with chronic conditions, the most common condition was chronic hypertension (31.1%), followed by thyroid disease (30.3%), allergy (28%), asthma (26.5%), and ophthalmologic disease (6.8%). Among the 161 participants who reported using regular medications, antihypertensive medications were the most common (34.8%), followed by thyroid hormones (27.3%), anti-asthmatics (18%), antihistamines (14.9%), and cholesterol-lowering drugs (13%).

The vast majority reported receiving a COVID-19 vaccination (91.2%), of whom 80.1% received three doses, 13.8% two doses, 4.5% four doses, and 1.6% one dose. About 38.4% of the participants received the influenza vaccine, of whom only 46.6% had received it within the last 12 months (Table 2).

### 3.3. HMPXV Information Sources

Only 25 participants (7.3%) reported learning about HMPXV within their undergraduate education. While the most commonly utilised sources of information about HMPXV were the Czech Ministry of Health (51.6%), digital news portals (47.5%), and social media (25.8%), the least commonly utilised sources were the U.S. CDC (1.5%), ECDC (5%), and scientific journals (5.6%). Utilising the U.S. CDC (*p* = 0.005), the WHO (*p* = 0.031), and scientific journals (*p* = 0.011) was significantly associated with a higher likelihood of HMPXV vaccine acceptance.

The mean number of information sources was 1.8 ± 1.2; this was significantly associated with the level of HMPXV vaccine acceptance (*p* = 0.006). The HMPXV vaccine-acceptant group utilised more information sources (2.5 ± 1.6) than the HMPXV vaccine-hesitant (1.9 ± 1.2) and HMPXV vaccine-rejecting (1.6 ± 1.0) groups (Table 3).

On evaluating confidence levels, the U.S. CDC had the highest mean confidence score of 6.0 ± 0.7 (range: 1–5), followed by scientific journals (5.8 ± 1.1) and the ECDC (5.8 ± 0.6). The least trustworthy sources were social media (3.6 ± 1.1), digital news portals (4.2 ± 1.1), and the Czech Ministry of Health (5.3 ± 1.1). The confidence levels of the Czech Ministry of Health (*p* = 0.026) and public health institutes (*p* = 0.033) were significantly associated with a higher likelihood of HMPXV vaccine-acceptance (Table 3).

### 3.4. HMPXV Perceived Knowledge

Perceived knowledge of HMPXV’s clinical presentation was the highest (3.1 ± 1.0); however, vaccination was the lowest domain (2.7 ± 1.0). None of the perceived knowledge domains was significantly associated with HMPXV vaccine acceptance (Table 4).

No demographic predictor was significantly associated with perceived knowledge scores, although females, homosexuals, older participants (>47 years old), divorced participants, and those providing clinical care to HMPXV cases tended to have higher overall perceived knowledge scores than males (14.6 ± 4.1 vs. 14.4 ± 5.0), heterosexuals (14.8 ± 1.5 vs. 14.6 ± 4.2), younger participants (14.7 ± 3.8 vs. 14.4 ± 4.5), single participants (15.2 ± 4.2 vs. 13.6 ± 4.5), and those who did not provide clinical care to HMPXV cases (15.6 ± 5.3 vs. 14.5 ± 4.1). The differences between medical vs. allied HCWs and participants with vs. without minors were statistically significant and clinically negligible (Table S1).

The number of COVID-19 vaccine doses was significantly associated with the overall perceived knowledge score (*p* = 0.020). The participants who had received four COVID-19 vaccine doses had the highest perceived knowledge scores (16.5 ± 4.4), while those who had received only a single dose had the lowest perceived knowledge scores (11.0 ± 3.5). Similarly, receiving an influenza vaccine (15.3 ± 4.2 vs. 14.2 ± 4.1; *p* = 0.019) and learning about HMPXV during undergraduate education (17.8 ± 3.2 vs. 14.3 ± 4.1; *p* < 0.001) were significantly associated with higher perceived knowledge scores (Table S1).

### 3.5. HMPXV Factual Knowledge

The overall mean factual knowledge score was 9.4 ± 4.6 (0–20), and the highest score was achieved by the clinical presentation domain (3.0 ± 1.5 (0–5)), while the lowest score was for the treatment domain (0.9 ± 0.9 (0–4)). The domains scores of HMPXV’s clinical presentation (*p* = 0.047), risk factors (*p* = 0.014), and vaccination (*p* = 0.031) were significantly associated with HMPXV vaccine acceptance (Table 4).

In the domain of HMPXV’s epidemiology, all items received more than 50% correct answers. The item about HMPXV’s endemicity had the most correct answers (59.8%), while 40.8% of the participants declared that they did not know the case–fatality ratio of HMPXV.

In the domain of HMPXV’s clinical presentation, the characteristic feature of HMPXV compared with smallpox infection was selected correctly by only 40.5% of the participants, while 41.6% acknowledged that they did not know. When asked about the possible symptoms of HMPXV infection, skin and mucosal lesions were the most commonly selected answer (80.9%), followed by fever (80.4%), fatigue (56%), and headache (55.4%), while the least common was respiratory symptoms (15.8%). When asked about the possible locations of HMPXV infection, extremities were the most commonly selected answer (61.6%), followed by face and mouth (57.2%), chest (52.8%), and genitalia (43.1%), while the least common was anus (29.6%). Only 23.2% and 11.7% of the participants acknowledged that they did not know the locations and symptoms of HMPXV infection, respectively (Table S2).

In the domain of HMPXV risk factors, 76% of the participants selected “direct contact with monkeypox rash, scabs or body fluids” as a potential transmission source. Only 23.5% knew that vertical transmission of HMPXV from pregnant women to their foetuses was possible. While 68.9% of the participants knew that HMPXV transmission was possible between homo- and heterosexual partners, 6.5% thought that it was only possible among homosexual partners, and less than one-quarter (24.6%) were unaware of the possibility of sexual transmission (Table S2).

In the domain of HMPXV vaccination, the least correctly answered question was the one about the availability of HMPXV vaccines—only 33.7% knew there were available vaccines against HMPXV. Interestingly, almost half of the participants (49.6%) reported being unaware of the vaccine that provided cross-protection against HMPXV, and only 38.7% knew that it was the smallpox vaccine (Table S2).

The domain of HMPXV treatment received the lowest number of correct answers. Only 25.2% knew that there were effective drugs against HMPXV, and 1.5%, 3.8%, and 11.7% of the participants named cidofovir, brincidofovir, and tecovirimat as effective antivirals for HMPXV infection, respectively (Table S2).

On evaluating the predictors of HMPXV factual knowledge, gender, sexual orientation, having minors, providing care, chronic illnesses, medical treatments, and COVID-19 and influenza vaccination were not associated with the HMPXV factual knowledge scores. The younger participants (≤47 years old), medical professionals, those receiving four COVID-19 vaccine doses, and the undergraduate curriculum were significantly associated with higher factual knowledge scores than older participants (10.0 ± 4.6 vs. 8.9 ± 4.5; *p* = 0.013), allied HCWs (11.1 ± 4.7 vs. 9.2 ± 4.5; *p* = 0.021), a single COVID-19 vaccine (11.5 ± 4.6 vs. 9.4 ± 4.7; *p* = 0.008), and not learning about HMPXV within undergraduate education (13.2 ± 3.0 vs. 9.1 ± 4.5; *p* < 0.001), respectively (Table S3).

The U.S. CDC had the highest factual knowledge score (15.2 ± 2.3), followed by the ECDC (12.4 ± 4.6), the WHO (12.3 ± 3.2), and scientific journals (12.2 ± 2.7), while the lowest scores were achieved by social media (9.7 ± 4.7), digital news portals (9.7 ± 4.4), and the Czech Ministry of Health (10.3 ± 4.2) (Table S3).

### 3.6. Consistency of HMPXV Vaccine Knowledge

Non-parametric correlation analysis between the perceived and factual knowledge domains revealed that HMPXV’s clinical presentation (Spearman’s *rho* = 0.369) and risk factors (*rho* = 0.339) had the largest correlation coefficients (Table 5).

On the other hand, the HMPXV treatment domain had the lowest correlation coefficient (*rho* = 0.197). While the HMPXV treatment domain’s mean factual knowledge score was 0.9 ± 0.9 (0–4), its perceived knowledge mean score was 2.8 ± 1.0 (1–5). These findings indicate that the participants might overestimate their knowledge about HMPXV treatment.

### 3.7. HMPXV-Vaccine-Related Perceptions

The overall scores of all perception domains—e.g., perceived susceptibility and severity—were significantly associated with HMPXV vaccine acceptance.

Regarding perceived susceptibility, the participants’ susceptibility due to their occupation had the highest mean score (2.5 ± 1.0), while susceptibility due to their lifestyle and health status had the lowest score (2.0 ± 0.9). COVID-19 vaccination was the only predictor that was significantly associated with perceived susceptibility (*p* = 0.048) (Table 6).

On evaluating perceived severity, the fear of being very sick due to HMPXV infection led to the highest perceived score (3.3 ± 0.9). The overall perceived severity score (9.1 ± 2.2 (3–15)) was significantly (*p* < 0.001) higher than the perceived susceptibility score (6.6 ± 2.5 (3–15)). The participants who did not have minors (9.3 ± 2.2 vs. 8.7 ± 2.2; *p* = 0.012) and those who received a COVID-19 vaccine (9.2 ± 2.2 vs. 8.1 ± 2.1; *p* = 0.016) had significantly higher severity scores than their counterparts. The participants who utilised scientific journals had the highest severity scores (9.8 ± 2.5) (Table S4).

Protection from serious complications had the highest score in the perceived benefits domain (3.5 ± 0.8), with an overall score of 9.9 ± 2.1 (3–15). The perceived barriers related to HMPXV vaccine safety (*p* = 0.038) and effectiveness (*p* = 0.035) were significantly associated with HMPXV vaccine acceptance (Table 6).

Chronic illnesses (*p* = 0.014), medical treatments (*p* = 0.010), COVID-19 vaccination (*p* < 0.001), and influenza vaccination (*p* = 0.011) were significantly associated with higher scores of perceived benefits. The participants who received four doses of the COVID-19 vaccine had a significantly higher perceived benefits score (11.6 ± 2.2) than those who received a single dose (7.4 ± 4.3) (Table S4).

Reliable evidence on the effectiveness and safety of HMPXV vaccines had the highest score as a cue to action (3.4 ± 1.0). The overall score of the cues to action domain was the second highest after the perceived benefits domain. Medical professionals (*p* = 0.045), chronic illnesses (*p* = 0.021), medical treatments (*p* = 0.028), COVID-19 vaccination (*p* < 0.001), influenza vaccination (*p* = 0.003) and having no minors (*p* = 0.011) had significantly higher cues to action scores than their counterparts (Table S4).

### 3.8. Health Belief Model (HBM)

When asked about their intentions to receive an HMPXV vaccine, 153 rejected it (44.9%), 158 (46.3%) were hesitant, and only 30 (8.8%) declared their acceptance to receive HMPXV vaccine. The HMPXV vaccine acceptance level was moderately correlated with cues to action (*rho* = 0.569), perceived susceptibility (*rho* = 0.424), and perceived benefits (*rho* = 0.372). Perceived barriers were weakly yet negatively correlated with HMPXV vaccine acceptance (*rho* = −0.149) (Table 7).

**Figure 2:**
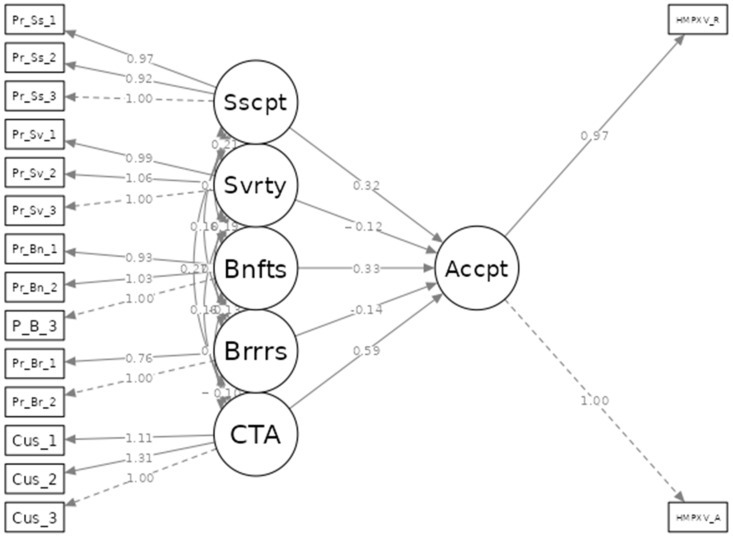
Structural equation model (SEM) of Czech HCWs’ perceptions and acceptance of HMPXV vaccines, September 2022 (*n* = 341). Sscpt = perceived susceptibility. Svrty = perceived severity. Bnfts = perceived benefits. Brrrs = perceived barriers. CTA = cues to action. Accpt = Acceptance. HMPXV vaccine_R = recommendation. HMPXV vaccine_A = acceptance.

Structural equation modelling (SEM) revealed that the proposed model had a good fit, as indicated by a mean square error of approximation (RMSEA) of 0.055 (CI 95%: 0.043–0.066), Tucker–Lewis index (TLI) of 0.992, and comparative fit index (CFI) of 0.994 (Figure 2).

### 3.9. Correlates to HMPXV Vaccine Acceptance

**Figure 3:**
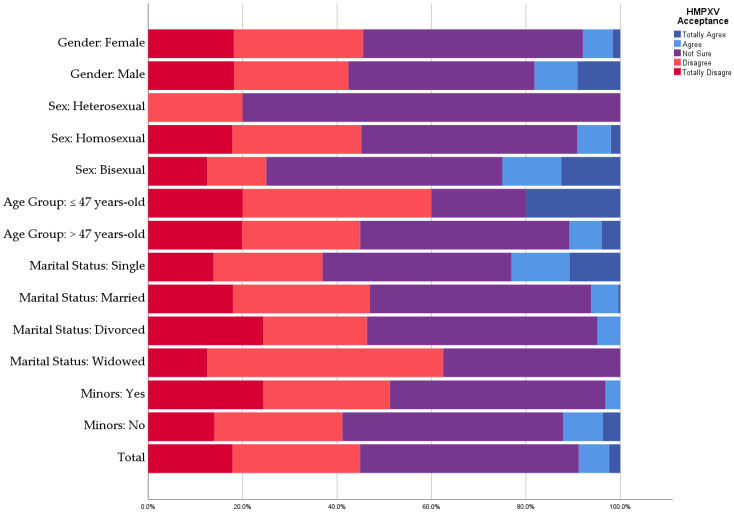
Demographic predictors of HMPXV vaccine acceptance among Czech HCWs, September 2022 (*n* = 341).

A total of 30 (8.8%) participants indicated their acceptance to receive an HMPXV vaccine, which represents a suboptimal vaccine acceptance—especially among HCWs. The HMPXV vaccine acceptance level was higher among males (18.2%) than females (7.9%). Heterosexual participants had the lowest HMPXV vaccine acceptance level (9.1%) compared with homosexuals (25%) and bisexuals (20%). Single participants had the highest HMPXV vaccine acceptance level (23.1%) compared with married (6.2%) and divorced (4.9%) participants. Conversely, having minors was significantly associated with a lower acceptance level (3.1% vs. 12.1%; *p* = 0.005). There were no significant differences between medical (11.1%) and allied HCWs (8.5%) in terms of HMPXV vaccine acceptance (Figure 3).

**Figure 4:**
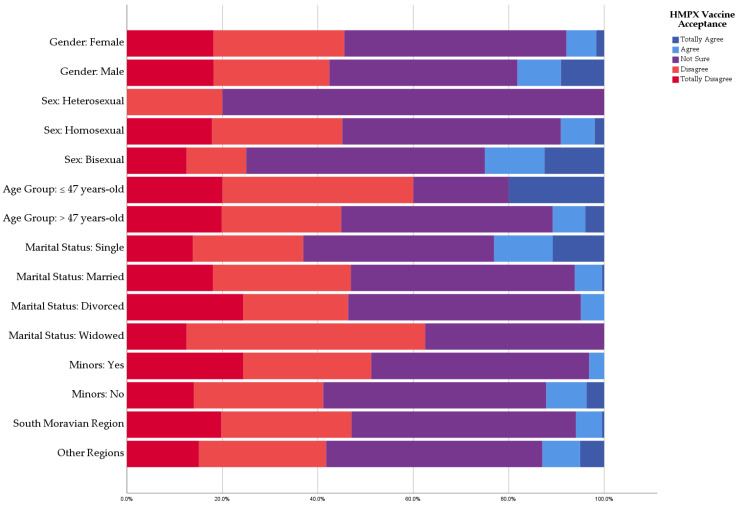
Anamnestic predictors of HMPXV vaccine acceptance among Czech HCWs, September 2022 (*n* = 341).

There were no statistically significant differences between the participants with/without chronic illness (8.3% vs. 9.1%; *p* = 0.810) or those with/without regular medications (9.9% vs. 7.8%; *p* = 0.482). All of the participants (100%) who did not receive a COVID-19 vaccine rejected the HMPXV vaccine, while 9.6% of COVID-19 vaccinees indicated their acceptance to receive an HMPXV vaccine. The number of COVID-19 vaccine doses was significantly (*p* = 0.041) associated with an increased likelihood of HMPXV vaccine acceptance (four doses: 28.6%, three doses: 10%; two doses: 2.3%; and single dose: 0%). Influenza vaccination was significantly associated with HMPXV vaccine acceptance (13% vs. 6.2%; *Sig* = 0.031) (Figure 4).

### 3.10. HMPXV Vaccine Recommendations and Willingness to Pay

Less than one-quarter (24.1%) of the participants agreed to recommend an HMPXV vaccine to their patients, family members, and friends—especially those at risk. The recommendation score was evaluated by a 5-point Likert scale ranging from “Strongly Disagree = 1” to “Strongly Agree = 1”. The mean recommendation score was significantly (*p* < 0.001) lower among the HMPXV vaccine-rejecting group (2.3 ± 1.0) compared with the vaccine-hesitant (3.2 ± 0.5) and vaccine-acceptant groups (4.3 ± 0.6) (Table 8).

Most participants (61%) suggested that an HMPXV vaccine should be offered to them for free, while 11.7% were willing to pay <10 EUR per shot, 22.9% agreed to pay 10–49 EUR per shot, and only 2.1% and 2.3% were willing to pay 50–99 EUR per shot and ≥100 EUR per shot, respectively. The largest proportion of the HMPXV vaccine-acceptant group (43.3%) agreed to pay 10–49 EUR per shot, followed by those who wanted it for free (40%) and those who were willing to pay ≥100 EUR per shot (10%). On the other hand, most of the HMPXV vaccine-rejecting group believed that the HMPXV vaccine should be free (Table 8).

Most participants (65.1%) suggested that the HMPXV vaccine should be offered to the public for free, while 17% suggested that 10–49 EUR per shot was a fair price, and 15.2% suggested <10 EUR per shot. The largest proportion of the HMPXV vaccine-acceptant group (65.7%) suggested that the public should be offered the HMPXV vaccine for free. Similarly, the largest proportion of the HMPXV vaccine-rejecting group (69.9%) suggested that the public should be offered the HMPXV vaccine for free (Table 8).

## 4. Discussion

Overall, the present study reveals several alarming findings about the levels of HMPXV-related knowledge and vaccine hesitancy among Czech HCWs. Only 8.8% of the participants agreed to receive a vaccination against HMPXV—44.9% rejected it, and 46.3% were hesitant. While digital news portals (47.5%) and social media (25.8%) were among the most utilised information sources about HMPXV, scientific journals (5.6%), the ECDC (5%), and the U.S. CDC (1.5%) were the least common sources. The participants demonstrated suboptimal levels of factual knowledge, especially regarding HMPXV vaccines (1.5 ± 1.2 (0–4)) and treatments (0.9 ± 0.9 (0–4)). Additionally, several misconceptions were detectable among the participants, such as the availability of effective vaccines and antivirals against HMPXV. The HBM indicated that the cues to action and perceived susceptibility were the most important constructs to predict HMPXV vaccine acceptance.

Since the outbreak of HMPXV in May 2022, a number of cross-sectional surveys have been conducted to evaluate the knowledge, perceptions, and attitudes of high-risk groups—including HCWs—regarding HMPXV and its vaccination [16,17,25,26,27,28,29,30,31,32,33]. Ricco et al. 2022 found that 58.6% of Italian physicians were in favour of receiving an HMPXV vaccine; nevertheless, they underestimated the risk of HMPXV as a pathogen compared to SARS-CoV-2, HIV, and TB [17]. In Saudi Arabia, Temsah et al. (2022) found that more than half of the general population was in favour of HMPXV vaccine implementation (50.6%), even though they were less worried about HMPXV compared to SARS-CoV-2 [30]. Moreover, Saudi HCWs believed that those who should be prioritised to receive HMPXV were HCWs themselves (69.8%), followed by immunocompromised patients (54.3%), the elderly (53.1%), and international travellers (40.4%) [16]. Interestingly, acceptance rates of HMPXV vaccines were significantly higher among men who have sex with men (MSM) [25,27]. In the Netherlands, 81.5% of the MSM surveyed by Dukers-Muijrers et al. in 2022 were willing to receive an HMPXV vaccine [27]. Similarly, most French MSM living with PrEP (79.3%) and HIV (59.8%) indicated their acceptance of HMPXV vaccination [25]. In our study, the level of HMPXV vaccine acceptance (8.8%) among Czech HCWs was not only lower than that of their counterparts in other countries [16,17] or high-risk individuals such as MSM [25,27], but also lower than that of the general population in the United States (46%) and Saudi Arabia (50.6%) [26,30].

The information sources utilised to learn about infectious disease outbreaks have a predictable impact on epidemic awareness and misinformation [34,35,36]. Therefore, Alshahrani et al. (2022) conducted a cross-sectional study to assess HMPXV-related knowledge among the general Saudi population, out of which 25% were HCWs [31]. Social media was the most utilised source among Saudis (75%), followed by TV and radio (45.6%), family members and friends (15.6%), and healthcare providers (13.8%), while only 8.8% of Saudis were reading scientific articles to learn about HMPXV [31]. In Saudi Arabia, the HMPXV-related knowledge score was low among 51.7% of social media users versus 21.4% of scientific article readers (*p* < 0.001) [31]. Another study among Saudi HCWs revealed that the most utilised sources of information were international health authorities (e.g., the WHO and U.S. CDC) (59.8%), followed by official local statements (57.6%), social media (51.1%), and scientific journals (24.5%) [16]. In Iraq, 62.2% used social media as their main source of information to learn about the HMPXV epidemic [32]. Our results differ slightly from the findings of these studies, as the Czech Ministry of Health (MoH) was the most commonly utilised information source (51.6%). Nevertheless, social media platforms were almost five times more common (25.8%) than scientific journals (5.6%), highlighting the systemic problem of the suboptimal practice of evidence-based medicine in the Czech Republic [37,38,39].

In the U.S., the general population rated HCWs as the most reliable information source about HMPXV, followed by public health institutions such as the CDC and the social media accounts of well-known physicians and researchers [26]. In an earlier study among the U.S. general population during the first wave of COVID-19, governmental information sources were the most reliable sources (e.g., CDC and FDA), followed by private media sources (e.g., CNN and FOX) and social media networks (e.g., Facebook and Twitter) [40]. Interestingly, trust in governmental sources was positively associated with better knowledge and protective behaviours. In contrast, trust in private media sources and social media was associated with less knowledge and protective behaviours [40]. In our study, the U.S. CDC received the highest evaluation by our participants (6.0 ± 0.7), followed by scientific journals (5.8 ± 1.1) and the ECDC (5.8 ± 0.6). These results should be interpreted with caution, because these were among our sample’s least commonly utilised sources.

Lipkus et al. (2013) found that factual knowledge about waterpipe tobacco smoking among college students was generally poor; however, their perceived knowledge was evaluated as average. As a result, knowledge gaps were suggested to exist among the surveyed students because of the weak correlation between factual knowledge and perceived knowledge [41]. Several studies among nurses revealed that the correlation between their factual knowledge and perceived knowledge with regard to diabetes mellitus was not strong, raising concerns about their competence in caring for diabetic patients [42,43,44,45]. A recent study exhibited that public health clinicians tended to overestimate their knowledge of research ethics guidelines because there was a mismatch between their high perceived knowledge and low factual knowledge [46]. One of the main consequences of the illusion of knowledge is undermining HCWs’ knowledge-seeking behaviours [46,47]. In our study, the correlation between perceived knowledge and factual knowledge with regard to HMPXV was not strong in any of the examined domains, indicating knowledge gaps among our participants [47,48]. While the mean HMPXV treatment factual knowledge score was 0.9 ± 0.9 (0–4), the mean HMPXV treatment perceived knowledge score was 2.8 ± 1.0 (1–5), and the correlation between them was weak (*rho* = 0.197).

On evaluating the predictors of HMPXV factual knowledge, gender, sexual orientation, having minors, providing care to HMPXV cases, chronic illnesses, medical treatments, and COVID-19 and influenza vaccination were not significantly associated with the HMPXV factual knowledge scores of our participants. Sallam et al. (2022) found that male HCWs and those with postgraduate degrees had higher HMPXV factual knowledge scores than females and those with undergraduate degrees in Jordan [29]. In Kuwait, Alsanafi et al. (2022) concluded that physicians had higher HMPXV factual knowledge scores than other HCWs—especially medical technicians and allied HCWs [28]. Among Kuwaiti HCWs, age, gender, income level, and education had no significant association with HMPXV factual knowledge [28]. In our study, the younger participants (≤47 years old) and medical professionals were significantly associated with higher factual knowledge scores compared to older participants (10.0 ± 4.6 vs. 8.9 ± 4.5; *p* = 0.013) and allied HCWs (11.1 ± 4.7 vs. 9.2 ± 4.5; *p* = 0.021), respectively.

The availability of effective vaccines (33.7%) and antivirals (25.2%) for HMPXV was among the least correctly answered factual knowledge items in the present study. Our results are comparable to those found among Jordanian HCWs, where only 33.3% were aware of the availability of effective vaccines [29]. In Italy, HCWs were more knowledgeable about the availability of effective HMPXV vaccines (60.1%) and antivirals (51.2%) [17].

Vertical transmission was one of the least correctly answered items by our participants (23.5%), indicating a potential knowledge gap concerning HMPXV-related pregnancy outcomes, which are known to be severe—including miscarriage (39% of pregnant cases), intrauterine foetal death (23%), and late foetal and perinatal loss (77%) [49]. A recent study among Saudi medical students found that only 36.5% were aware of the possibility of vertical transmission [33].

Another misconception revealed by our study was the proposition that HMPXV could be transmitted exclusively between homosexual partners (6.5%). In Jordan, 58.7% of HCWs thought that MSM had a role in spreading HMPXV, and this misconception was associated with other conspiracy beliefs about the epidemic’s origins [29].

The HBM demonstrated that cues to action were the strongest predictor of HMPXV vaccine acceptance (*rho* = 0.569) in our study, followed by perceived susceptibility (*rho* = 0.424) and perceived benefits (*rho* = 0.372). Similarly, Temsah et al. (2022) found that those who perceived HMPXV as dangerous and virulent had higher odds (OR: 1.456; CI 95%: 1.165–1.820) of accepting the HMPXV vaccine [30]. In Italy, HCWs with a higher perceived risk of HMPXV exhibited favourable attitudes towards the HMPXV vaccine [17]. While 34%, 56.6%, and 37.5% of our participants thought the HMPXV vaccine could protect against natural infection, prevent serious complications, and protect their families and patients, respectively, our participants perceived lower benefits of the HMPXV vaccine than Italian physicians, as most Italians believed that the HMPXV vaccine could prevent natural infection (90.2%) and severe complications (90.8%) [17].

Amid the COVID-19 pandemic, the HBM was extensively used to explain and predict COVID-19 vaccine acceptance [50,51,52]. In Hong Kong, perceived severity, perceived benefits, and cues to action were positively correlated with COVID-19 vaccine acceptance; however, perceived susceptibility had no significant association with vaccine acceptance [50]. Among Chinese pregnant women, cues to action were the strongest predictor for COVID-19 vaccine acceptance, followed by perceived susceptibility and perceived benefits [51]. Moreover, among Chinese HCWs, cues to action, perceived severity, and perceived benefits were positively correlated with COVID-19 vaccine acceptance, while perceived barriers and perceived susceptibility were not associated with acceptance [52].

The availability of reliable scientific evidence on HMPXV vaccine effectiveness and safety as a cue to action was significantly (*p* < 0.001) different among the HMPXV vaccine-rejecting (3.0 ± 1.1), -hesitant (3.7 ± 0.8), and -acceptant (4.2 ± 0.9) groups. In an earlier study among Czech HCWs, the safety and effectiveness of COVID-19 vaccine booster doses were significantly associated with the boosters’ acceptance [53]. Similarly, COVID-19 vaccine boosters’ safety was a strong predictor of acceptance among German university staff and students [54], Polish HCWs and healthcare students [55], and Algerian HCWs [56]. In the present study, perceived barriers—such as concerns regarding HMPXV vaccines’ safety (*p* = 0.038) and effectiveness (*p* = 0.035)—were significantly associated with HMPXV vaccine acceptance.

Regarding predictors of HMPXV vaccine acceptance, males (18.2%), homosexuals (25%), and single participants (23.1%) had higher acceptance levels than females (7.9%), heterosexuals (9.1%), and married participants (6.2%). In the U.S. general population, the female gender was associated with lower odds of HMPXV vaccine acceptance (OR: 0.42; CI 95%: 0.31–0.58) compared with males [26]. Nevertheless, no statistically significant difference among Italian physicians was found between males and females [17].

In our study, receiving COVID-19 vaccination (*p* < 0.001), number of COVID-19 vaccine doses (*p* = 0.023), receiving seasonal influenza vaccination (*p* < 0.001), and recent administration of seasonal influenza vaccination (*p* = 0.002) were significantly associated with HMPXV vaccine acceptance. These results are consistent with what was found among the U.S. general population, where COVID-19 vaccinees had higher odds of HMPXV vaccine acceptance (OR: 29.61; CI 95%: 15.68–55.91) compared with non-vaccinees [26]. Additionally, French MSM with PrEP who received COVID-19 vaccines were significantly more associated with higher odds of HMPXV vaccine acceptance than their counterparts who were not vaccinated against COVID-19 [25].

The low level of HMPXV vaccine acceptance (8.8%) among our participants should also be viewed in the wider context of Czech HCWs’ attitudes towards vaccination. In a 2018 European Commission (EC) report on vaccine confidence in Europe, the Czech Republic was the only country where HCWs had lower confidence levels in the safety and importance of the MMR vaccine than the general public [57]. Additionally, 29% of Czech and 19% of Slovak HCWs did not think that seasonal influenza vaccines were important, and 36.4% of Czech and 24.8% of Slovak HCWs did not think that seasonal influenza vaccines were safe [57]. Czech general physicians (GPs) were the least interested in recommending seasonal influenza vaccines to their patients (25.2%) compared to all other European GPs, including Estonian (65%), French (83%), German (87%), Italian (87%), Polish (49%), and Spanish (93%) GPs [57].

### 4.1. Strengths

To the best of our knowledge, this is the first theory-based study to examine HMPXV-related knowledge and readiness to receive the HMPXV vaccine among HCWs. The HBM is an extensively used model in studying health-related behaviours, including preventive behaviours such as vaccine acceptance [58,59]. Another strength of the present study is the use of two distinct knowledge constructs—i.e., perceived knowledge and factual knowledge—in order to point out knowledge gaps through inconsistency between the two constructs [48]. The factual knowledge items revealed critical misconceptions among the surveyed population, such as the availability of effective vaccines and antivirals, the risk of vertical transmission, and homosexual stigmatisation.

### 4.2. Limitations

The first limitation of our study is the gender imbalance in the recruited sample, as most of the participants were females (88.9%); however, this is not far from the reality of the target population, as most Czech HCWs are females (80.2%) [60]. The second limitation is the underrepresentation of medical professionals (i.e., physicians, dentists, and pharmacists), who represent 22.3% of the target population, while in our study they were only 10.6% of the entire sample [60]. The third limitation is the non-random sampling technique that we used in this study, which may induce self-selection and reporting biases, as those interested in HMPXV, infectious diseases, or public health emergencies were more likely to join our participants. The fourth limitation is the geographical distribution of the sample; considering that the highest number of respondents were from South Moravia and the fact that the research team was based in South Moravia, there may have been a higher motivation for respondents to answer with respect to the group of HCWs collaborating with the second largest university and medical faculty in the Czech Republic.

### 4.3. Implications

The findings of this study call upon public health practitioners and health policymakers in the Czech Republic to act accordingly in order to determine the drivers of vaccine hesitancy among Czech HCWs. The low frequency of using scientific journals to learn about infectious diseases (5.6%), compared with the high reliance on news portals (47.5%) and social media (25.8%), highlights another systemic problem—the unfamiliarity of evidence-based practice culture within the Czech healthcare system. The overestimated perceived knowledge of Czech HCWs and their poor factual knowledge—especially with regard to HMPXV vaccines and treatments—indicate a critical and common issue of the illusion of knowledge among the target population. Dedicated educational campaigns should address the knowledge gaps with regard to the availability of effective HMPXV vaccines and treatments, the risk of vertical transmission, and homosexual stigmatisation. Future studies should investigate the prevalence and drivers of HMPXV vaccine hesitancy among the general population to scout for potential misinformation and its sources.

## 5. Conclusions

The present study reveals several alarming findings about the levels of HMPXV-related knowledge and vaccine hesitancy among Czech HCWs. Only 8.8% of the participants agreed to receive vaccination against HMPXV, 44.9% rejected it, and 46.3% were hesitant. While digital news portals (47.5%) and social media (25.8%) were among the most utilised sources of information about HMPXV, scientific journals (5.6%), the ECDC (5%), and the U.S. CDC (1.5%) were the least common sources. The weak correlation between participants’ perceived knowledge and factual knowledge—especially concerning HMPXV vaccines and treatments—confirms the possibility of knowledge gaps. Dedicated educational campaigns should address the knowledge gaps with respect to the availability of effective HMPXV vaccines and treatments, the risk of vertical transmission, and homosexual stigmatisation.

## Figures and Tables

**Table 1:** Demographic characteristics of Czech HCWs responding to the HMPXV survey, September 2022 (*n* = 341).

Variable	Outcome	HMPXV Vaccine Rejection (*n* = 153)	HMPXV Vaccine Hesitancy (*n* = 158)	HMPXV Vaccine Acceptance (*n* = 30)	Total (*n* = 341)	*p*
Gender	Female	138 (90.2%)	141 (89.2%)	24 (80.0%)	303 (88.9%)	0.266
	Male	14 (9.2%)	13 (8.2%)	6 (20.0%)	33 (9.7%)	0.161
	I prefer not to say	1 (0.7%)	4 (2.5%)	0 (0%)	5 (1.5%)	0.488
Sexual Orientation	Heterosexual	134 (87.6%)	136 (86.1%)	27 (90%)	297 (87.1%)	0.859
	Homosexual	2 (1.3%)	4 (2.5%)	2 (6.7%)	8 (2.3%)	0.188
	Bisexual	3 (2%)	1 (0.6%)	1 (3.3%)	5 (1.5%)	0.214
	I prefer not to say	14 (14%)	17 (10.8%)	0 (0%)	31 (9.1%)	0.157
Age	*µ* ± *SD*	45.9 ± 11.3	47.6 ± 12.0	39.9 ± 13.9	46.1 ± 12.0	0.026
Marital Status	Single	24 (15.7%)	26 (16.5%)	15 (50%)	65 (19.1%)	<0.001
	Married	99 (64.7%)	99 (62.7%)	13 (43.3%)	211 (61.9%)	0.085
	Divorced	19 (12.4%)	20 (12.7%)	2 (6.7%)	41 (12%)	0.772
	Widow	5 (3.3%)	3 (1.9%)	0 (0%)	8 (2.3%)	0.554
	I prefer not to say	6 (3.9%)	10 (6.3%)	0 (0%)	16 (4.7%)	0.340
Having Minors	Yes	65 (42.5%)	58 (36.7%)	4 (13.3%)	127 (37.2%)	0.010
	No	88 (57.5%)	100 (63.3%)	26 (86.7%)	214 (62.8%)	
Location	South Moravian Region	95 (62.1%)	95 (60.1%)	12 (40%)	202 (59.2%)	0.076
	Other regions	58 (37.9%)	63 (39.9%)	18 (60%)	139 (40.8%)	
Profession	Medical	11 (%)	21 (%)	4 (13.3%)	36 (10.6%)	0.163
	Allied HCWs	142 (92.8%)	137 (86.7%)	26 (86.7%)	305 (89.4%)	
Providing Care	Yes	6 (3.9%)	8 (5.1%)	2 (6.7%)	16 (4.7%)	0.689
	No	147 (96.1%)	150 (94.9%)	28 (93.3%)	325 (95.3%)	

The chi-squared (*χ*^2^) test, Fisher’s exact test, and the Kruskal–Wallis (*H*) test were used with a significance level (*p*) of ≤0.05.

**Table 2:** Anamnestic characteristics of Czech HCWs responding to the HMPXV survey, September 2022 (*n* = 341).

Variable	Outcome	HMPXV Vaccine Rejection (*n* = 153)	HMPXV Vaccine Hesitancy (*n* = 158)	HMPXV Vaccine Acceptance (*n* = 30)	Total (*n* = 341)	*p*
	Total ^†^	58 (37.9%)	63 (39.9%)	11 (36.7%)	132 (38.7%)	0.912
^†^ Chronic Illnesses	Allergy	20 (34.5%)	13 (20.6%)	4 (36.4%)	37 (28%)	0.179
	Asthma	16 (27.6%)	15 (23.8%)	4 (36.4%)	35 (26.5%)	0.634
	Blood disease	3 (5.2%)	2 (3.2%)	0 (0%)	5 (3.8%)	0.788
	Bowel disease	3 (5.2%)	3 (4.8%)	0 (0%)	6 (4.5%)	1.000
	Cancer	0 (0%)	4 (6.3%)	0 (0%)	4 (3.0%)	0.183
	Cardiovascular disease	1 (1.7%)	3 (4.8%)	1 (9.1%)	5 (3.8%)	0.280
	Chronic hypertension	19 (32.8%)	20 (31.7%)	2 (18.2%)	41 (31.1%)	0.718
	COPD *	1 (1.7%)	2 (3.2%)	1 (9.1%)	4 (3.0%)	0.381
	Diabetes mellitus I	0 (0%)	1 (1.6%)	0 (0%)	1 (0.8%)	1.000
	Diabetes mellitus II	4 (6.9%)	2 (3.2%)	0 (0%)	6 (4.5%)	0.662
	Liver disease	1 (1.7%)	1 (1.6%)	2 (18.2%)	4 (3.0%)	0.035
	Psychological distress	3 (5.2%)	6 (9.5%)	4 (36.4%)	13 (9.8%)	0.016
	Neurological disorder	3 (5.2%)	7 (11.1%)	3 (27.3%)	13 (9.8%)	0.071
	Ophthalmologic disease	4 (6.9%)	5 (7.9%)	0 (0%)	9 (6.8%)	1.000
	Renal disease	0 (0%)	1 (1.6%)	1 (9.1%)	2 (1.5%)	0.160
	Rheumatoid arthritis	3 (5.2%)	2 (3.2%)	0 (0%)	5 (3.8%)	0.788
	Thyroid disease	18 (31.0%)	18 (28.6%)	4 (36.4%)	40 (30.3%)	0.846
	Other	6 (10.3%)	9 (14.3%)	2 (18.2%)	17 (12.9%)	0.624
^‡^ Medications	Total ^‡^	70 (45.8%)	75 (47.5%)	16 (53.3%)	161 (47.2%)	0.760
	Anti-asthmatics	8 (11.4%)	16 (21.3%)	5 (31.3%)	29 (18.0%)	0.099
	Anticoagulants	3 (4.3%)	2 (2.7%)	0 (0%)	5 (3.1%)	0.807
	Antidepressants	5 (7.1%)	6 (8.0%)	6 (37.5%)	17 (10.6%)	0.006
	Antidiabetics	4 (5.7%)	3 (4.0%)	0 (0%)	7 (4.3%)	0.864
	Antiepileptics	1 (1.4%)	1 (1.3%)	1 (6.3%)	3 (1.9%)	0.450
	Antihistamines	15 (21.4%)	8 (10.7%)	1 (6.3%)	24 (14.9%)	0.139
	Antihypertensives	27 (38.6%)	26 (34.7%)	3 (18.8%)	56 (34.8%)	0.324
	Anti-reflux	5 (7.1%)	6 (8.0%)	3 (18.8%)	14 (8.7%)	0.295
	Immunosuppressants	2 (2.9%)	4 (5.3%)	0 (0%)	6 (3.7%)	0.832
	Cholesterol-lowering	8 (11.4%)	11 (14.7%)	2 (12.5%)	21 (13.0%)	0.887
	Common analgesics	1 (1.4%)	4 (5.3%)	3 (18.8%)	8 (5.0%)	0.027
	Contraceptives	9 (12.9%)	7 (9.3%)	2 (12.5%)	18 (11.2%)	0.709
	Corticosteroids	2 (2.9%)	2 (2.7%)	1 (6.3%)	5 (3.1%)	0.628
	NSAIDs	3 (4.3%)	4 (5.3%)	1 (6.3%)	8 (5.0%)	0.881
	Opioid analgesics	1 (1.4%)	0 (0%)	0 (0%)	1 (0.6%)	0.534
	Thyroid hormones	17 (24.3%)	24 (32.0%)	3 (18.8%)	44 (27.3%)	0.467
	Other	8 (11.4%)	11 (14.7%)	4 (25.0%)	23 (14.3%)	0.324
COVID-19 Vaccine	Yes Ϯ	129 (84.3%)	152 (96.2%)	30 (100.0%)	311 (91.2%)	<0.001
	No	24 (15.7%)	6 (3.8%)	0 (0%)	30 (8.8%)	
Ϯ COVID-19 Vaccine Doses	One dose	3 (2.3%)	2 (1.3%)	0 (0%)	5 (1.6%)	0.798
	Two doses	21 (16.3%)	21 (13.8%)	1 (3.3%)	43 (13.8%)	0.167
	Three doses	103 (79.8%)	121 (79.6%)	25 (83.3%)	249 (80.1%)	0.894
	Four doses	2 (1.6%)	8 (5.3%)	4 (13.3%)	14 (4.5%)	0.020
Influenza Vaccine	Yes ^ψ^	43 (28.1%)	71 (44.9%)	17 (56.7%)	131 (38.4%)	<0.001
	No	110 (71.9%)	87 (55.1%)	13 (43.3%)	210 (61.6%)	
^ψ^ Last 12 Months	Yes	11 (25.6%)	40 (56.3%)	10 (58.8%)	61 (46.6%)	0.003
	No	32 (74.4%)	31 (43.7%)	7 (41.2%)	70 (53.4%)	

The chi-squared (*χ*^2^) test and Fisher’s exact test were used with a significance level (*p*) of ≤0.05. * COPD = chronic obstructive pulmonary disease. ^†^ refers to chronic illnesses. ^‡^ refers to medications. ^Ϯ^ refers to COVID-19 vaccinees. ^ψ^ refers to seasonal influenza vaccinees.

**Table 3:** Information sources utilised by Czech HCWs responding to the HMPXV survey, September 2022 (*n* = 341).

Variable	Outcome	HMPXV Vaccine Rejection (*n* = 153)	HMPXV Vaccine Hesitancy (*n* = 158)	HMPXV Vaccine Acceptance (*n* = 30)	Total (*n* = 341)	*p.*
Undergrad Curriculum	Yes	8 (5.2%)	13 (8.2%)	4 (13.3%)	25 (7.3%)	0.216
	No	145 (94.8%)	145 (91.8%)	26 (86.7%)	316 (92.7%)	
Utilised Sources	Ministry of Health (MZČR)	70 (45.8%)	91 (57.6%)	15 (50.0%)	176 (51.6%)	0.111
	Public health institutes (e.g., ÚZIS and SZÚ) *	18 (11.8%)	30 (19.0%)	6 (20.0%)	54 (15.8%)	0.166
	European Centres for Disease Prevention and Control (ECDC)	5 (3.3%)	9 (5.7%)	3 (10.0%)	17 (5.0%)	0.186
	U.S. Centers for Disease Control and Prevention (CDC)	1 (0.7%)	1 (0.6%)	3 (10.0%)	5 (1.5%)	0.005
	World Health Organization (WHO)	20 (13.1%)	25 (15.8%)	10 (33.3%)	55 (16.1%)	0.031
	Professional medical associations	16 (10.5%)	21 (13.3%)	4 (13.3%)	41 (12.0%)	0.704
	Scientific journals	4 (2.6%)	10 (6.3%)	5 (16.7%)	19 (5.6%)	0.011
	Social media (e.g., Facebook and Twitter)	39 (25.5%)	41 (25.9%)	8 (26.7%)	88 (25.8%)	0.989
	News portals (e.g., iDNES and BLESK) **	70 (45.8%)	74 (46.8%)	18 (60.0%)	162 (47.5%)	0.351
	Other	3 (2.0%)	5 (3.2%)	2 (6.7%)	10 (2.9%)	0.279
	Total (*µ* ± *SD*)	1.6 ± 1.0	1.9 ± 1.2	2.5 ± 1.6	1.8 ± 1.2	0.006
Confidence Level (1–7)	Ministry of Health (MZČR)	5.1 ± 1.1	5.4 ± 1.0	5.7 ± 1.0	5.3 ± 1.1	0.026
	Public health institutes (e.g., ÚZIS and SZÚ)	5.4 ± 0.9	5.8 ± 0.8	6.3 ± 0.5	5.7 ± 0.8	0.033
	European Centres for Disease Prevention and Control (ECDC)	6.0 ± 0.0	5.9 ± 0.8	5.3 ± 0.6	5.8 ± 0.6	0.288
	U.S. Centers for Disease Control and Prevention (CDC)	6.0 ± 0.0	7.0 ± 0.0	5.7 ± 0.6	6.0 ± 0.7	0.333
	World Health Organization (WHO)	5.6 ± 0.8	5.7 ± 0.7	6.1 ± 0.9	5.7 ± 0.8	0.112
	Professional medical associations	5.6 ± 0.8	5.5 ± 0.8	6.5 ± 0.6	5.6 ± 0.8	0.057
	Scientific journals	4.8 ± 1.5	6.0 ± 0.7	6.4 ± 0.9	5.8 ± 1.1	0.119
	Social media (e.g., Facebook and Twitter)	3.9 ± 1.0	3.5 ± 1.1	3.0 ± 1.4	3.6 ± 1.1	0.110
	News portals (e.g., iDNES and BLESK)	4.3 ± 0.9	4.2 ± 1.1	4.1 ± 1.5	4.2 ± 1.1	0.813

The chi-squared (*χ*^2^) test, Fisher’s exact test, analysis of variance (ANOVA), and the Kruskal–Wallis (*H*) test were used with a significance level (*p*) of ≤0.05. * ÚZIS is the Institute of Health Information and Statistics of the Czech Republic (IHIS-CR). SZÚ is the National Institute of Public Health (NIPH). ** iDNES and BLESK are tabloid newspapers.

**Table 4:** Knowledge of Czech HCWs responding to the HMPXV survey, September 2022 (*n* = 341).

Variable	Outcome	HMPXV Vaccine Rejection (*n* = 153)	HMPXV Vaccine Hesitancy (*n* = 158)	HMPXV Vaccine Acceptance (*n* = 30)	Total (*n* = 341)	*p*
Perceived Knowledge	Epidemiology: (1–5)	2.7 ± 0.9	2.9 ± 0.9	2.9 ± 1.0	2.8 ± 1.0	0.053
	Clinical presentation: (1–5)	3.0 ± 1.0	3.1 ± 1.0	3.2 ± 1.0	3.1 ± 1.0	0.250
	Risk factors: (1–5)	3.2 ± 1.0	3.3 ± 0.9	3.4 ± 0.9	3.2 ± 1.0	0.532
	Vaccination: (1–5)	2.6 ± 1.0	2.8 ± 0.9	2.8 ± 1.0	2.7 ± 1.0	0.316
	Treatment: (1–5)	2.7 ± 1.0	2.9 ± 0.9	2.8 ± 1.1	2.8 ± 1.0	0.094
Factual Knowledge: Epidemiology	Incubation period	95 (62.1%)	90 (57.0%)	18 (60.0%)	203 (59.5%)	0.653
	Case–fatality ratio	76 (49.7%)	76 (48.1%)	20 (66.7%)	172 (50.4%)	0.170
	Endemic region	99 (64.7%)	89 (56.3%)	16 (53.3%)	204 (59.8%)	0.241
	Total (0–3)	1.8 ± 1.1	1.6 ± 1.1	1.8 ± 1.2	1.7 ± 1.1	0.425
Factual Knowledge: Clinical Presentation	Clinical symptoms	135 (88.2%)	138 (87.3%)	29 (96.7%)	302 (88.6%)	0.387
	Differential diagnosis	62 (40.5%)	59 (37.3%)	17 (56.7%)	138 (40.5%)	0.142
	Lesions’ locations	118 (77.1%)	119 (75.3%)	25 (83.3%)	262 (76.8%)	0.630
	Total (0–5)	3.0 ± 1.5	3.0 ± 1.6	3.7 ± 1.4	3.0 ± 1.6	0.047
Factual Knowledge: Risk Factors	Transmission pathways	127 (83.0%)	131 (82.9%)	28 (93.3%)	286 (83.9%)	0.372
	Vertical transmission	34 (22.2%)	33 (20.9%)	13 (43.3%)	80 (23.5%)	0.026
	Sexual transmission	109 (71.2%)	101 (63.9%)	25 (83.3%)	235 (68.9%)	0.077
	Total (0–4)	2.3 ± 1.1	2.2 ± 1.2	2.8 ± 1.0	2.3 ± 1.2	0.014
Factual Knowledge: Vaccination	Vaccine availability	54 (35.3%)	44 (27.8%)	17 (56.7%)	115 (33.7%)	0.008
	Pre-exposure prophylaxis	88 (57.5%)	95 (60.1%)	20 (66.7%)	203 (59.5%)	0.633
	Cross-immunisation	58 (37.9%)	59 (37.3%)	15 (50.0%)	132 (38.7%)	0.411
	Total (0–4)	1.5 ± 1.2	1.5 ± 1.3	2.1 ± 1.2	1.5 ± 1.2	0.031
Factual Knowledge: Treatment	Treatment availability	36 (23.5%)	43 (27.2%)	7 (23.3%)	86 (25.2%)	0.733
	Medications listed	18 (11.8%)	27 (17.1%)	6 (20.0%)	51 (15.0%)	0.257
	Prognosis	75 (49.0%)	75 (47.5%)	15 (50.0%)	165 (48.4%)	0.947
	Total (0–4)	0.9 ± 0.9	0.9 ± 0.9	0.9 ± 0.7	0.9 ± 0.9	0.640

The chi-squared (χ^2^) test, Fisher’s exact test, and the Kruskal–Wallis (*H*) test were used with a significance level (*p*) of ≤0.05.

**Table 5:** Correlation between perceived and factual knowledge of Czech HCWs responding to the HMPXV survey, September 2022 (*n* = 341).

			Perceived Knowledge				
			Epidemiology	Clinical Presentation	Risk Factors	Vaccination	Treatment
**Factual Knowledge**	**Epidemiology**	*rho*	0.295	0.287	0.227	0.235	0.160
		*p*	<0.001	<0.001	<0.001	<0.001	<0.001
	**Clinical** **Presentation**	*rho*	0.369	0.369	0.303	0.289	0.253
		*p*	<0.001	<0.001	<0.001	<0.001	<0.001
	**Risk Factors**	*rho*	0.390	0.383	0.339	0.294	0.223
		*p*	<0.001	<0.001	<0.001	<0.001	<0.001
	**Vaccination**	*rho*	0.289	0.277	0.194	0.270	0.173
		*p*	<0.001	<0.001	<0.001	<0.001	<0.001
	**Treatment**	*rho*	0.295	0.334	0.201	0.261	0.197
		*p*	<0.001	<0.001	<0.001	<0.001	<0.001

Non-parametric correlation (Spearman’s *rho*) was used, with a significance level (*p*) of ≤0.05.

**Table 6:** HMPXV-vaccine-related perceptions of Czech HCWs responding to the HMPXV survey, September 2022 (*n* = 341).

Category	Item	HMPXV Vaccine Rejection (*n* = 153)	HMPXV Vaccine Hesitancy (*n* = 158)	HMPXV Vaccine Acceptance (*n* = 30)	Total (*n* = 341)	*p*
Perceived Susceptibility	1. Due to my occupation.	2.0 ± 0.9	2.7 ± 0.9	3.4 ± 1.2	2.5 ± 1.0	<0.001
	2. Due to lifestyle and health status.	1.7 ± 0.9	2.2 ± 0.8	2.5 ± 1.2	2.0 ± 0.9	<0.001
	3. Not vaccinated vs. smallpox.	1.8 ± 0.9	2.4 ± 0.8	2.9 ± 1.3	2.2 ± 1.0	<0.001
	Overall score (3–15)	5.6 ± 2.3	7.2 ± 2.1	8.8 ± 2.9	6.6 ± 2.5	<0.001
Perceived Severity	1. I will be very sick.	3.2 ± 1.0	3.3 ± 0.8	3.8 ± 1.0	3.3 ± 0.9	0.004
	2. I may require hospitalisation.	2.8 ± 1.0	2.9 ± 0.7	3.2 ± 1.3	2.9 ± 0.9	0.195
	3. I might die.	2.9 ± 1.0	3.0 ± 0.8	3.4 ± 1.1	3.0 ± 0.9	0.130
	Overall score (3–15)	8.9 ± 2.4	9.1 ± 1.8	10.3 ± 2.9	9.1 ± 2.2	0.011
Perceived Benefits	1. Protected from getting infected.	3.0 ± 0.8	3.3 ± 0.7	3.8 ± 0.8	3.2 ± 0.8	<0.001
	2. Protected from serious complications.	3.3 ± 0.8	3.6 ± 0.6	4.4 ± 0.7	3.5 ± 0.8	<0.001
	3. Protect my patients and family.	2.9 ± 1.0	3.3 ± 0.7	4.1 ± 0.9	3.2 ± 0.9	<0.001
	Overall score (3–15)	9.1 ± 2.3	10.1 ± 1.6	12.2 ± 2.0	9.9 ± 2.1	<0.001
Perceived Barriers	1. Safety of HMPXV vaccine.	3.1 ± 1.0	3.0 ± 0.7	2.7 ± 1.3	3.0 ± 0.9	0.038
	2. Effectiveness of HMPXV vaccine.	3.0 ± 1.0	2.9 ± 0.7	2.6 ± 1.3	2.9 ± 0.9	0.035
	Overall score (2–10)	6.1 ± 1.9	5.9 ± 1.3	5.3 ± 2.6	6.0 ± 1.7	0.015
Cues to Action	1. Mandated by the employer.	2.4 ± 1.0	3.3 ± 0.8	3.5 ± 1.5	2.9 ± 1.1	<0.001
	2. Recommended by health authorities.	2.4 ± 1.0	3.3 ± 0.8	3.9 ± 1.2	3.0 ± 1.0	<0.001
	3. Reliable evidence on effectiveness and safety.	3.0 ± 1.1	3.7 ± 0.8	4.2 ± 0.9	3.4 ± 1.0	<0.001
	Overall score (3–15)	7.9 ± 2.6	10.3 ± 1.9	11.6 ± 2.9	9.3 ± 2.7	<0.001

The Kruskal–Wallis (*H*) test was used, with a significance level (*p*) of ≤0.05.

**Table 7:** Correlation between HMPXV vaccine perceptions and acceptance of Czech HCWs responding to the HMPXV survey, September 2022 (*n* = 341).

		Perceived Susceptibility	Perceived Severity	Perceived Benefits	Perceived Barriers	Cues to Action	Acceptance
**Perceived** **Susceptibility**	*rho*	1.000	0.203	0.114	0.103	0.307	0.424
	*p*		<0.001	<0.001	0.058	<0.001	<0.001
**Perceived** **Severity**	*rho*	0.203	1.000	0.174	0.092	0.209	0.145
	*p*	<0.001		<0.001	0.088	<0.001	0.007
**Perceived** **Benefits**	*rho*	0.114	0.174	1.000	−0.178	0.393	0.372
	*p*	<0.001	<0.001		<0.001	<0.001	<0.001
**Perceived** **Barriers**	*rho*	0.103	0.092	−0.178	1.000	−0.090	−0.149
	*p*	0.058	0.088	<0.001		0.099	0.006
**Cues to Action**	*rho*	0.307	0.209	0.393	−0.090	1.000	0.569
	*p*	<0.001	<0.001	<0.001	0.099		<0.001
**Acceptance**	*rho*	0.424	0.145	0.372	−0.149	0.569	1.000
	*p*	<0.001	0.007	<0.001	0.006	<0.001	

Non-parametric correlation (Spearman’s *rho*) was used, with a significance level (*p*) of ≤0.05.

**Table 8:** HMPXV vaccine recommendations and willingness to Pay (WTP) of Czech HCWs responding to the HMPXV survey, September 2022 (*n* = 341).

Variable	Outcome	HMPXV Vaccine Rejection (*n* = 153)	HMPXV Vaccine Hesitancy (*n* = 158)	HMPXV Vaccine Acceptance (*n* = 30)	Total (*n* = 341)	*p*
I am willing/interested in recommending a monkeypox vaccination to my patients, family members, and friends—especially those at risk.	Strongly disagree	39 (25.5%)	0 (0%)	0 (0%)	39 (11.4%)	<0.001
	Disagree	57 (37.3%)	4 (2.5%)	0 (0%)	61 (17.9%)	<0.001
	Not sure	37 (24.2%)	120 (75.9%)	2 (6.7%)	159 (46.6%)	<0.001
	Agree	19 (12.4%)	31 (19.6%)	18 (60.0%)	68 (19.9%)	<0.001
	Strongly agree	1 (0.7%)	3 (1.9%)	10 (33.3%)	14 (4.1%)	<0.001
	Total (*µ* ± *SD*)	2.3 ± 1.0	3.2 ± 0.5	4.3 ± 0.6	2.9 ± 1.0	<0.001
How much would you like to pay for a human monkeypox vaccine shot as a personal expense?	It should be free	106 (69.3%)	90 (57.0%)	12 (40.0%)	208 (61.0%)	0.004
	<10 EUR/shot	15 (9.8%)	24 (15.2%)	1 (3.3%)	40 (11.7%)	0.128
	10–49 EUR/shot	26 (17.0%)	39 (24.7%)	13 (43.3%)	78 (22.9%)	0.005
	50–99 EUR/shot	3 (2.0%)	3 (1.9%)	1 (3.3%)	7 (2.1%)	0.712
	≥100 EUR/shot	3 (2.0%)	2 (1.3%)	3 (10.0%)	8 (2.3%)	0.031
What is the optimal price of the human monkeypox vaccine for the public?	It should be free	107 (69.9%)	98 (62.0%)	17 (56.7%)	222 (65.1%)	0.205
	<10 EUR/shot	14 (9.2%)	30 (19.0%)	8 (26.7%)	52 (15.2%)	0.008
	10–49 EUR/shot	25 (16.3%)	29 (18.4%)	4 (13.3%)	58 (17.0%)	0.764
	50–99 EUR/shot	3 (2.0%)	1 (0.6%)	1 (3.3%)	5 (1.5%)	0.214
	≥100 EUR/shot	4 (2.6%)	0 (0%)	0 (0%)	4 (1.2%)	0.147

The chi-squared (*χ*^2^) test, Fisher’s exact test, and the Kruskal–Wallis (*H*) test were used with a significance level (*p*) of ≤0.05.

## Data Availability

The data that support the findings of this study are available from the corresponding author upon reasonable request.

## Supplementary Materials

The following supporting information can be downloaded at: https://www.mdpi.com/article/10.3390/vaccines10122022/s1, Figure S1: Sample size calculation via Epi-Info^TM^; Population Survey module (StatCalc). Table S1: Predictors of HMPXV-related Perceived Knowledge of Czech Healthcare Workers Responding to HMPXV Survey; Table S2: Factual Knowledge of Czech Healthcare Workers Responding to HMPXV Survey; Table S3: Predictors of HMPXV-related Factual Knowledge of Czech Healthcare Workers Responding to HMPXV Survey. Table S4: Predictors of HMPXV vaccine-related Perceptions and Acceptance of Czech Healthcare Workers Responding to HMPXV Survey.
