# Supplementary material for: Monkeypox Knowledge and Vaccine Hesitancy of Czech Healthcare Workers: A Health Belief Model (HBM)-Based Study
Source: Vaccines (Basel). 2022 Nov 26;10(12):2022. doi: 10.3390/vaccines10122022 (PMC9788212; doi:10.3390/vaccines10122022)
Supplement: Supplementary file 1 [file vaccines-10-02022-s001.zip › vaccines-2020356-supplementary.pdf]

Figure S1. Sample size calculation via Epi-Info™; Population Survey module (StatCalc).

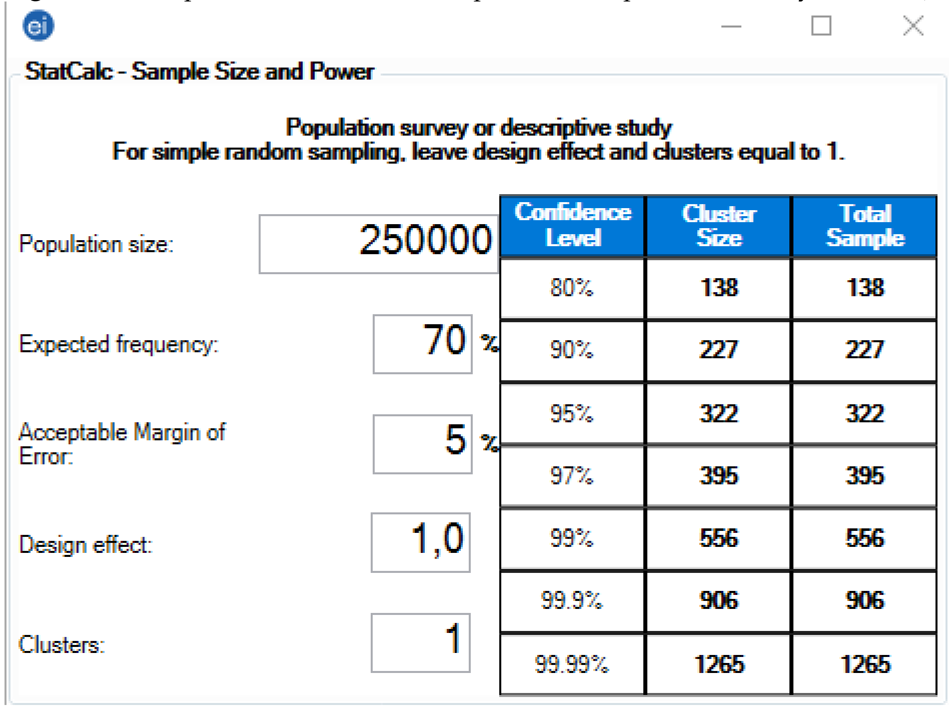

Table S1. Predictors of HMPXV-related Perceived Knowledge of Czech Healthcare Workers Responding to HMPXV vaccine Survey, September 2022, (n = 341)

Variable	Outcome	Epidemiology	*Sig.*	Clinical Presentation	*Sig.*	Risk Factors	*Sig.*	Vaccination	*Sig.*	Treatment	*Sig.*	Total	*Sig.*
Gender	Female	2.8 ± 0.9	0.966	3.1 ± 1.0	0.936	3.2 ± 0.9	0.855	2.8 ± 0.9	0.487	2.8 ± 0.9	0.921	14.6 ± 4.1	0.972
	Male	2.8 ± 1.1		3.0 ± 1.2		3.2 ± 1.2		2.6 ± 1.1		2.8 ± 1.2		14.4 ± 5.0	
	Prefer not to say	2.8 ± 1.3		2.8 ± 1.3		3.0 ± 1.4		2.8 ± 1.3		2.6 ± 1.1		14.0 ± 6.3	
Sexual Orientation	Heterosexual	2.8 ± 0.9	0.351	3.1 ± 1.0	0.992	3.2 ± 1.0	0.629	2.7 ± 1.0	0.635	2.8 ± 1.0	0.672	14.6 ± 4.2	0.930
	Homosexual	2.8 ± 0.5		3.1 ± 0.8		3.6 ± 0.5		2.6 ± 0.7		2.6 ± 0.7		14.8 ± 1.5	
	Bisexual	3.4 ± 0.9		3.0 ± 1.2		3.0 ± 1.2		2.6 ± 1.1		2.4 ± 1.1		14.4 ± 5.1	
	Prefer not to say	2.9 ± 0.9		3.1 ± 1.1		3.2 ± 1.1		2.9 ± 1.0		2.8 ± 1.0		14.9 ± 4.6	

Age Group	≤ 47 years-old	2.8 ± 1.0	0.523	3.1 ± 1.1	0.474	3.2 ± 1.1	0.809	2.6 ± 1.0	0.033	2.7 ± 1.0	0.249	14.4 ± 4.5	0.681
	> 47 years-old	2.8 ± 0.8		3.0 ± 0.9		3.2 ± 0.9		2.8 ± 0.9		2.9 ± 0.9		14.7 ± 3.8	
Marital Status	Single	2.7 ± 1.0	0.513	2.9 ± 1.1	0.541	3.0 ± 1.1	0.190	2.5 ± 1.0	0.057	2.5 ± 1.1	0.035	13.6 ± 4.5	0.203
	Married	2.8 ± 0.9		3.1 ± 0.9		3.3 ± 0.9		2.8 ± 0.9		2.9 ± 0.9		14.9 ± 3.9	
	Divorced	2.8 ± 1.0		3.0 ± 0.9		3.4 ± 0.9		3.0 ± 1.0		3.0 ± 0.9		15.2 ± 4.2	
	Widow	2.4 ± 0.9		2.6 ± 1.2		2.8 ± 1.3		2.4 ± 0.9		2.4 ± 1.1		12.5 ± 5.2	
	Prefer not to say	2.9 ± 1.1		3.1 ± 1.1		3.1 ± 1.1		2.8 ± 0.9		2.8 ± 0.9		14.6 ± 4.8	
Having Minors	Yes	2.8 ± 1.0	0.720	3.1 ± 1.1	0.650	3.2 ± 1.1	0.753	2.8 ± 1.0	0.665	2.8 ± 1.0	0.763	14.7 ± 4.6	0.471
	No	2.8 ± 0.9		3.0 ± 0.9		3.2 ± 0.9		2.7 ± 0.9		2.8 ± 0.9		14.5 ± 3.9	
Location	South Moravian Region	2.8 ± 0.9	0.669	3.1 ± 1.0	0.980	3.3 ± 0.9	0.060	2.8 ± 0.9	0.023	2.8 ± 0.9	0.300	14.8 ± 4.0	0.177
	Other Regions	2.8 ± 1.0		3.1 ± 1.0		3.1 ± 1.0		2.6 ± 1.0		2.7 ± 1.0		14.3 ± 4.4	
Profession	Medical	2.8 ± 1.0	0.816	3.2 ± 1.1	0.346	3.2 ± 1.1	0.827	2.6 ± 1.2	0.568	2.7 ± 1.1	0.688	14.5 ± 4.8	0.945
	Allied HCPs	2.8 ± 0.9		3.0 ± 1.0		3.2 ± 1.0		2.7 ± 0.9		2.8 ± 0.9		14.6 ± 4.1	
Providing Care	Yes	3.1 ± 1.1	0.142	3.4 ± 1.2	0.102	3.5 ± 1.2	0.160	2.8 ± 1.2	0.991	2.8 ± 1.0	0.994	15.6 ± 5.3	0.336
	No	2.8 ± 0.9		3.0 ± 1.0		3.2 ± 1.0		2.7 ± 0.9		2.8 ± 1.0		14.5 ± 4.1	
Chronic Illnesses	Yes	2.8 ± 0.9	0.349	3.0 ± 0.9	0.663	3.1 ± 1.0	0.085	2.8 ± 0.9	0.349	2.8 ± 0.9	0.611	14.5 ± 4.3	0.695
	No	2.8 ± 0.9		3.1 ± 1.0		3.3 ± 1.0		2.7 ± 1.0		2.8 ± 1.0		14.6 ± 4.1	
Medical Treatments	Yes	2.7 ± 0.9	0.302	3.0 ± 0.9	0.421	3.2 ± 0.9	0.150	2.7 ± 0.9	0.459	2.8 ± 0.9	0.632	14.3 ± 4.1	0.247
	No	2.9 ± 0.9		3.1 ± 1.0		3.3 ± 1.0		2.8 ± 1.0		2.8 ± 1.0		14.8 ± 4.3	
COVID-19 Vaccination	Yes	2.8 ± 0.9	0.299	3.1 ± 1.0	0.169	3.3 ± 1.0	0.103	2.7 ± 1.0	0.537	2.8 ± 1.0	0.999	14.7 ± 4.2	0.294
	No	2.6 ± 0.8		2.8 ± 0.8		3.0 ± 0.9		2.6 ± 0.9		2.8 ± 0.9		13.8 ± 3.7	
COVID-19 Vaccine Doses	One Dose	2.2 ± 1.1	0.141	2.8 ± 0.8	0.613	2.6 ± 1.1	0.219	1.8 ± 0.8	0.039	1.6 ± 0.9	0.004	11.0 ± 3.5	0.020
	Two Doses	3.0 ± 1.0		3.2 ± 1.1		3.4 ± 1.0		2.9 ± 1.0		3.0 ± 1.0		15.3 ± 4.3	
	Three Doses	2.8 ± 0.9		3.1 ± 1.0		3.2 ± 1.0		2.7 ± 0.9		2.8 ± 0.9		14.5 ± 4.2	
	Four Doses	3.1 ± 0.9		3.3 ± 1.0		3.6 ± 0.8		3.1 ± 1.1		3.4 ± 1.1		16.5 ± 4.4	
Influenza Vaccination	Yes	3.0 ± 0.9	<0.001	3.3 ± 1.0	0.006	3.3 ± 0.9	0.244	2.8 ± 1.0	0.569	2.9 ± 1.0	0.090	15.3 ± 4.2	0.019
	No	2.7 ± 0.9		2.9 ± 1.0		3.2 ± 1.0		2.7 ± 1.0		2.7 ± 0.9		14.2 ± 4.1	
Last 12 Months	Yes	3.1 ± 0.9	0.313	3.4 ± 1.0	0.061	3.5 ± 0.9	0.019	3.0 ± 1.0	0.087	3.1 ± 1.0	0.128	16.1 ± 4.1	0.054
	No	2.9 ± 0.9		3.1 ± 0.9		3.2 ± 0.9		2.6 ± 0.9		2.8 ± 0.9		14.6 ± 4.1	
Undergrad Curriculum	Yes	3.5 ± 0.8	<0.001	3.8 ± 0.8	<0.001	3.9 ± 0.8	<0.001	3.2 ± 0.8	0.010	3.4 ± 0.7	<0.001	17.8 ± 3.2	<0.001
	No	2.7 ± 0.9		3.0 ± 1.0		3.2 ± 1.0		2.7 ± 1.0		2.8 ± 1.0		14.3 ± 4.1	
Utilised Sources	Ministry of Health	3.0 ± 0.9	<0.001	3.2 ± 0.9	<0.001	3.4 ± 0.8	0.017	2.9 ± 0.9	0.005	2.9 ± 0.9	0.002	15.4 ± 3.7	<0.001
	Public Health Institutes	3.2 ± 0.8	<0.001	3.5 ± 0.9	<0.001	3.7 ± 0.9	<0.001	3.1 ± 0.9	0.004	3.2 ± 1.0	<0.001	16.6 ± 3.7	<0.001
	ECDC	3.5 ± 1.1	0.003	3.5 ± 1.0	0.044	3.6 ± 1.0	0.117	3.2 ± 1.0	0.036	2.9 ± 1.0	0.631	16.8 ± 4.5	0.036

US CDC	4.0 ± 1.0	0.011	4.2 ± 0.8	0.011	4.2 ± 0.8	0.024	4.0 ± 1.0	0.011	3.6 ± 1.1	0.088	20.0 ± 4.5	0.015
WHO	3.2 ± 1.0	0.002	3.5 ± 0.9	0.001	3.6 ± 0.9	<0.001	3.1 ± 0.9	0.005	3.1 ± 0.9	0.023	16.4 ± 3.8	<0.001
Professional Associations	3.1 ± 0.9	0.016	3.3 ± 1.0	0.043	3.4 ± 1.0	0.154	2.9 ± 1.0	0.436	3.0 ± 0.9	0.110	15.8 ± 4.2	0.057
Scientific Journals	3.1 ± 0.8	0.358	3.3 ± 0.8	0.363	3.4 ± 0.8	0.540	3.0 ± 1.2	0.381	3.2 ± 1.0	0.132	16.0 ± 4.4	0.261
Social Media	2.7 ± 0.9	0.494	3.0 ± 0.9	0.686	3.4 ± 0.8	0.048	2.7 ± 0.9	0.607	2.8 ± 0.9	0.803	14.6 ± 3.5	0.886
News Portals	2.8 ± 0.8	0.694	3.0 ± 0.9	0.548	3.3 ± 0.9	0.710	2.8 ± 0.9	0.075	2.9 ± 0.9	0.127	14.8 ± 3.9	0.506
Overall Score: μ ± SD (min-max)	2.8 ± 0.9 (1 – 5)		3.1 ± 1.0 (1 – 5)		3.2 ± 1.0 (1 – 5)		2.7 ± 1.0 (1 – 5)		2.8 ± 1.0 (1 – 5)		14.6 ± 4.2 (5 – 25)	

Kruskal-Wallis (H) test and Mann-Whitney (U) test were used with a significance level (*Sig.*) of ≤ 0.05. 4

Table S2. Factual Knowledge of Czech Healthcare Workers Responding to HMPXV Survey, September 2022, (*n* = 341) 5

	Question	Answer	HMPXV Vaccine Rejection (*n* = 153)	HMPXV Vaccine Hesitancy (*n* = 158)	HMPXV Vaccine Acceptance (*n* = 30)	Total (*n* = 341)	*Sig.*
Epidemiology	The incubation period of human monkeypox ranges between …	3 – 7 days	11 (7.2%)	18 (11.4%)	4 (13.3%)	33 (9.7%)	0.293
		7 – 21 days	95 (62.1%)	90 (57.0%)	18 (60.0%)	203 (59.5%)	0.653
		21 – 28 days	2 (1.3%)	6 (3.8%)	2 (6.7%)	10 (2.9%)	0.151
		I do not know	45 (29.4%)	44 (27.8%)	6 (20.0%)	95 (27.9%)	0.575
	The case-fatality ratio of human monkeypox usually ranges between …	4% and 11%	76 (49.7%)	76 (48.1%)	20 (66.7%)	172 (50.4%)	0.170
		14% and 19%	7 (4.6%)	9 (5.7%)	2 (6.7%)	18 (5.3%)	0.764
		20% and 30%	5 (3.3%)	7 (4.4%)	0 (0%)	12 (3.5%)	0.681
		I do not know	65 (42.5%)	66 (41.8%)	8 (26.7%)	139 (40.8%)	0.256
	Monkeypox used to be endemic in …	southeast Asia	8 (5.2%)	13 (8.2%)	3 (10.0%)	24 (7.0%)	0.429
		sub-Saharan Africa	99 (64.7%)	89 (56.3%)	16 (53.3%)	204 (59.8%)	0.241
		north America	1 (0.7%)	3 (1.9%)	0 (0%)	4 (1.2%)	0.739
		I do not know	45 (29.4%)	53 (33.5%)	11 (36.7%)	109 (32.0%)	0.624
Clinical Presentation	Which of the following are symptoms of monkeypox infection? (*select all that apply*)	Fever	124 (81.0%)	122 (77.2%)	28 (93.3%)	274 (80.4%)	0.120
		Chills	75 (49.0%)	71 (44.9%)	19 (63.3%)	165 (48.4%)	0.177
		Headache	92 (60.1%)	78 (49.4%)	19 (63.3%)	189 (55.4%)	0.107
		Myalgia	69 (45.1%)	64 (40.5%)	16 (53.3%)	149 (43.7%)	0.385
		Lymphadenopathy	71 (46.4%)	67 (42.4%)	18 (60.0%)	156 (45.7%)	0.203
		Fatigue	89 (58.2%)	80 (50.6%)	22 (73.3%)	191 (56.0%)	0.055
		Respiratory symptoms	23 (15.0%)	21 (13.3%)	10 (33.3%)	54 (15.8%)	0.034

		Skin and mucosal lesions	121 (79.1%)	128 (81.0%)	27 (90.0%)	276 (80.9%)	0.394
		I do not know	19 (12.4%)	20 (12.7%)	1 (3.3%)	40 (11.7%)	0.383
	… is/are the diagnostic feature(s) of monkeypox that distinguish(es) it from smallpox.	Lymphadenopathy	62 (40.5%)	59 (37.3%)	17 (56.7%)	138 (40.5%)	0.142
		Fever	5 (3.3%)	4 (2.5%)	0 (0%)	9 (2.6%)	0.784
		Respiratory symptoms	24 (15.7%)	25 (15.8%)	3 (10.0%)	52 (15.2%)	0.810
		I do not know	62 (40.5%)	70 (44.3%)	10 (33.3%)	142 (41.6%)	0.499
	The possible locations to find monkeypox lesions are … *(select all that apply)*	Genitalia	70 (45.8%)	61 (38.6%)	16 (53.3%)	147 (43.1%)	0.221
		Anus	41 (26.8%)	47 (29.7%)	13 (43.3%)	101 (29.6%)	0.193
		Extremities	92 (60.1%)	97 (61.4%)	21 (70.0%)	210 (61.6%)	0.595
		Chest	75 (49.0%)	83 (52.5%)	22 (73.3%)	180 (52.8%)	0.051
		Back	69 (45.1%)	78 (49.4%)	21 (70.0%)	168 (49.3%)	0.045
		Face and Mouth	80 (52.3%)	96 (60.8%)	19 (63.3%)	195 (57.2%)	0.248
		I do not know	35 (22.9%)	39 (24.7%)	5 (16.7%)	79 (23.2%)	0.630
**Risk Factors**	Human monkeypox can be transmitted through … *(select all that apply)*	contact with respiratory secretions.	64 (41.8%)	61 (38.6%)	16 (53.3%)	141 (41.3%)	0.320
		touching objects and fabrics used by someone with monkeypox.	49 (32.0%)	69 (43.7%)	19 (63.3%)	137 (40.2%)	0.003
		direct contact with monkeypox rash, scabs or body fluids.	117 (76.5%)	116 (73.4%)	26 (86.7%)	259 (76.0%)	0.292
		I do not know	26 (17.0%)	27 (17.1%)	2 (6.7%)	55 (16.1%)	0.372
	Vertical transmission, from a pregnant person to their fetus through placenta, of human monkeypox is possible.	True	34 (22.2%)	33 (20.9%)	13 (43.3%)	80 (23.5%)	0.026
		False	17 (11.1%)	16 (10.1%)	1 (3.3%)	34 (10.0%)	0.505
		I do not know	102 (66.7%)	109 (69.0%)	16 (53.3%)	227 (66.6%)	0.249
	Sexual transmission of human monkeypox is possible among …	homosexual partners only.	9 (5.9%)	13 (8.2%)	0 (0%)	22 (6.5%)	0.271
		heterosexual partners only.	0 (0%)	0 (0%)	0 (0%)	0 (0%)	N/A
		both of homo- and heterosexual partners.	109 (71.2%)	101 (63.9%)	25 (83.3%)	235 (68.9%)	0.077
		I do not know	35 (22.9%)	44 (27.8%)	5 (16.7%)	84 (24.6%)	0.340
**Vaccination**	An effective vaccine against human monkeypox is to date available.	Ture	54 (35.3%)	44 (27.8%)	17 (56.7%)	115 (33.7%)	0.008
		False	28 (18.3%)	41 (25.9%)	6 (20.0%)	75 (22.0%)	0.256
		I do not know	71 (46.4%)	73 (46.2%)	7 (23.3%)	151 (44.3%)	0.054
	Pre-exposure Prophylaxis (PrEP) of monkeypox … *(select all that apply)*	should be administered to healthcare professionals caring for monkeypox patients.	55 (35.9%)	65 (41.1%)	15 (50.0%)	135 (39.6%)	0.306

		should be administered to laboratory workers dealing with orthopoxviruses samples and cultures.	36 (23.5%)	41 (25.9%)	12 (40.0%)	89 (26.1%)	0.171
		should be administered to individuals with close contact / exposure to monkeypox.	58 (37.9%)	61 (38.6%)	15 (50.0%)	134 (39.3%)	0.450
		can prevent onset of the disease, if administered until four days after exposure.	16 (10.5%)	21 (13.3%)	7 (23.3%)	44 (12.9%)	0.156
		I do not know	65 (42.5%)	63 (39.9 %)	10 (33.3%)	138 (40.5%)	0.633
	Which vaccine(s) provide(s) cross-immunization against human monkeypox?	Chickenpox Vaccine	5 (3.3%)	7 (4.4%)	2 (6.7%)	14 (4.1%)	0.507
		Smallpox Vaccine	58 (37.9%)	59 (37.3%)	15 (50.0%)	132 (38.7%)	0.411
		COVID-19 Vaccine	0 (0%)	1 (0.6%)	2 (6.7%)	3 (0.9%)	0.021
		None of the above	12 (7.8%)	8 (5.1%)	3 (10.0%)	23 (6.7%)	0.438
		I do not know	78 (51.0%)	83 (52.5%)	8 (26.7%)	169 (49.6%)	0.031
**Treatment**	Effective drugs against human monkeypox are to date available.	Ture	36 (23.5%)	43 (27.2%)	7 (23.3%)	86 (25.2%)	0.733
		False	39 (25.5%)	28 (17.7%)	13 (43.3%)	80 (23.5%)	0.007
		I do not know	78 (51.0%)	87 (55.1%)	10 (33.3%)	175 (51.3%)	0.092
	For severe monkeypox cases, … can be used. *(select all that apply)*	tecovirimat	16 (10.5%)	21 (13.3%)	3 (10.0%)	40 (11.7%)	0.745
		brincidofovir	1 (0.7%)	3 (1.9%)	1 (3.3%)	5 (1.5%)	0.293
		cidofovir	3 (2.0%)	8 (5.1%)	2 (6.7%)	13 (3.8%)	0.202
		I do not know	135 (88.2%)	131 (82.9%)	24 (80.0%)	290 (85.0%)	0.257
	For most cases, monkeypox virus infection leads to …	mild and self-limiting clinical course.	75 (49.0%)	75 (47.5%)	15 (50.0%)	165 (48.4%)	0.947
		moderate yet life-threatening clinical course.	43 (28.1%)	39 (24.7%)	7 (23.3%)	89 (26.1%)	0.740
		fatal and life-threatening clinical course.	7 (4.6%)	5 (3.2%)	3 (10.0%)	15 (4.4%)	0.159
		I do not know	28 (18.3%)	39 (24.7%)	5 (16.7%)	72 (21.1%)	0.318

Chi-squared (χ^2) test and Fisher's exact test were used with a significance level (*Sig.*) of ≤ 0.05.

Table S3. Predictors of HMPXV-related Factual Knowledge of Czech Healthcare Workers Responding to HMPXV Survey, September 2022, (n = 341)

Variable	Outcome	Epidemiology	*Sig.*	Clinical Presentation	*Sig.*	Risk Factors	*Sig.*	Vaccination	*Sig.*	Treatment	*Sig.*	Total	*Sig.*
Gender	Female	1.7 ± 1.1	0.564	3.1 ± 1.6	0.345	2.3 ± 1.2	0.517	1.5 ± 1.2	0.806	0.9 ± 0.9	0.669	9.4 ± 4.5	0.916
	Male	1.8 ± 1.2		3.1 ± 1.7		2.1 ± 1.2		1.6 ± 1.2		0.9 ± 1.1		9.4 ± 5.3	
	Prefer not to say	2.2 ± 0.9		2.4 ± 0.6		2.0 ± 0.7		1.6 ± 0.9		1.0 ± 1.0		9.2 ± 2.3	
Sexual Orientation	Heterosexual	1.7 ± 1.1	0.694	3.0 ± 1.5	0.798	2.3 ± 1.2	0.181	1.5 ± 1.2	0.892	0.9 ± 0.9	0.470	9.5 ± 4.6	0.785
	Homosexual	2.1 ± 0.8		3.3 ± 1.3		2.9 ± 0.6		1.4 ± 1.3		0.8 ± 0.7		10.4 ± 3.3	
	Bisexual	1.4 ± 1.3		3.4 ± 1.9		2.0 ± 1.2		1.2 ± 1.3		0.6 ± 0.9		8.6 ± 5.8	
	Prefer not to say	1.7 ± 1.1		2.9 ± 1.4		2.0 ± 1.1		1.5 ± 1.2		0.7 ± 0.7		8.7 ± 4.4	
Age Group	≤ 47 years-old	1.8 ± 1.1	0.107	3.2 ± 1.5	0.006	2.4 ± 1.2	0.004	1.6 ± 1.2	0.305	0.9 ± 0.9	0.618	10.0 ± 4.6	0.013
	> 47 years-old	1.6 ± 1.1		2.8 ± 1.5		2.1 ± 1.1		1.5 ± 1.2		0.9 ± 0.9		8.9 ± 4.5	
Marital Status	Single	1.6 ± 1.1	0.647	3.3 ± 1.5	0.060	2.5 ± 1.3	0.028	1.6 ± 1.1	0.432	0.8 ± 0.8	0.281	9.8 ± 4.4	0.161
	Married	1.7 ± 1.1		3.1 ± 1.5		2.3 ± 1.1		1.5 ± 1.3		1.0 ± 0.9		9.6 ± 4.5	
	Divorced	1.8 ± 1.1		2.8 ± 1.6		2.2 ± 1.1		1.3 ± 1.2		0.7 ± 0.8		8.7 ± 4.9	
	Widow	1.1 ± 1.2		1.8 ± 1.9		1.1 ± 1.4		1.1 ± 0.8		1.0 ± 0.8		6.1 ± 4.4	
	Prefer not to say	1.8 ± 1.1		2.6 ± 1.5		1.9 ± 1.1		1.8 ± 1.3		0.9 ± 0.8		9.0 ± 4.9	
Having Minors	Yes	1.8 ± 1.2	0.132	3.2 ± 1.5	0.196	2.4 ± 1.2	0.165	1.6 ± 1.3	0.306	1.0 ± 0.9	0.212	9.9 ± 4.8	0.064
	No	1.6 ± 1.1		3.0 ± 1.5		2.2 ± 1.1		1.5 ± 1.2		0.9 ± 0.9		9.1 ± 4.4	
Location	South Moravian Region	1.7 ± 1.1	0.670	3.0 ± 1.5	0.357	2.2 ± 1.2	0.244	1.5 ± 1.2	0.238	0.9 ± 0.9	0.532	9.2 ± 4.7	0.478
	Other Regions	1.7 ± 1.1		3.1 ± 1.5		2.4 ± 1.1		1.6 ± 1.3		0.9 ± 0.9		9.7 ± 4.3	
Profession	Medical	1.9 ± 1.1	0.346	3.3 ± 1.6	0.199	2.5 ± 1.1	0.274	2.1 ± 1.3	0.005	1.4 ± 1.2	0.006	11.1 ± 4.7	0.021
	Allied HCPs	1.7 ± 1.1		3.0 ± 1.5		2.3 ± 1.2		1.5 ± 1.2		0.8 ± 0.8		9.2 ± 4.5	
Providing Care	Yes	1.8 ± 1.1	0.681	3.4 ± 1.7	0.149	2.6 ± 1.4	0.158	1.7 ± 1.4	0.667	1.1 ± 1.2	0.785	10.6 ± 5.7	0.201
	No	1.7 ± 1.1		3.0 ± 1.5		2.3 ± 1.2		1.5 ± 1.3		0.9 ± 0.9		9.4 ± 4.5	
Chronic Illnesses	Yes	1.7 ± 1.1	0.672	3.0 ± 1.5	0.604	2.2 ± 1.2	0.384	1.6 ± 1.2	0.409	0.9 ± 0.8	0.998	9.4 ± 4.5	0.882
	No	1.7 ± 1.1		3.1 ± 1.5		2.3 ± 1.2		1.5 ± 1.3		0.9 ± 0.9		9.5 ± 4.6	
Medical Treatments	Yes	1.6 ± 1.1	0.162	2.9 ± 1.6	0.216	2.2 ± 1.2	0.120	1.5 ± 1.3	0.931	0.8 ± 0.9	0.233	9.1 ± 4.6	0.177
	No	1.8 ± 1.1		3.1 ± 1.5		2.4 ± 1.2		1.5 ± 1.2		1.0 ± 0.9		9.7 ± 4.5	
COVID-19 Vaccination	Yes	1.7 ± 1.1	0.183	3.1 ± 1.5	0.777	2.3 ± 1.2	0.852	1.6 ± 1.2	0.112	0.9 ± 0.9	0.396	9.5 ± 4.6	0.758
	No	2.0 ± 1.0		3.0 ± 1.5		2.2 ± 1.2		1.2 ± 1.1		1.0 ± 0.8		9.3 ± 4.1	
COVID-19 Vaccine Doses	One Dose	1.4 ± 1.5	0.109	3.4 ± 1.5	0.009	2.6 ± 1.3	0.001	1.6 ± 1.5	0.323	0.4 ± 0.5	0.382	9.4 ± 4.7	0.008
	Two Doses	1.9 ± 1.1		3.6 ± 1.4		2.9 ± 1.1		1.7 ± 1.2		1.1 ± 1.0		11.1 ± 4.0	

	Three Doses	1.6 ± 1.1		2.9 ± 1.5		2.2 ± 1.2		1.5 ± 1.2		0.9 ± 0.9		9.0 ± 4.6	
	Four Doses	2.1 ± 1.1		3.7 ± 1.7		2.6 ± 1.1		2.0 ± 1.2		1.0 ± 0.9		11.5 ± 4.6	
Influenza Vaccination	Yes	1.7 ± 1.1	0.871	2.9 ± 1.6	0.343	2.2 ± 1.1	0.173	1.7 ± 1.3	0.069	0.9 ± 0.9	0.792	9.4 ± 4.7	0.774
	No	1.7 ± 1.1		3.1 ± 1.5		2.3 ± 1.2		1.4 ± 1.2		0.9 ± 0.9		9.5 ± 4.5	
Last 12 Months	Yes	1.8 ± 1.1	0.181	3.3 ± 1.5	0.018	2.3 ± 1.0	0.658	1.8 ± 1.3	0.378	1.1 ± 1.0	0.059	10.2 ± 4.7	0.088
	No	1.6 ± 1.1		2.6 ± 1.6		2.1 ± 1.2		1.6 ± 1.3		0.7 ± 0.8		8.7 ± 4.6	
Undergrad Curriculum	Yes	2.6 ± 0.8	<0.001	3.8 ± 1.0	0.013	3.0 ± 0.8	0.001	2.7 ± 0.9	<0.001	1.2 ± 1.2	0.351	13.2 ± 3.0	<0.001
	No	1.6 ± 1.1		3.0 ± 1.5		2.2 ± 1.2		1.4 ± 1.2		0.9 ± 0.9		9.1 ± 4.5	
Utilised Sources	Ministry of Health	1.9 ± 1.0	0.012	3.3 ± 1.3	0.001	2.5 ± 1.1	0.001	1.7 ± 1.3	0.042	1.0 ± 0.9	0.277	10.3 ± 4.2	<0.001
	Public Health Institutes	2.2 ± 0.9	0.001	3.7 ± 1.2	0.002	2.6 ± 1.0	0.105	2.0 ± 1.3	0.002	1.2 ± 1.0	0.046	11.5 ± 3.8	<0.001
	ECDC	2.1 ± 1.1	0.102	3.7 ± 1.4	0.080	2.9 ± 1.1	0.012	2.2 ± 1.4	0.028	1.5 ± 1.2	0.031	12.4 ± 4.6	0.006
	US CDC	2.8 ± 0.5	0.020	4.4 ± 0.9	0.031	3.2 ± 0.5	0.052	3.2 ± 0.9	0.005	1.6 ± 0.9	0.074	15.2 ± 2.3	0.003
	WHO	2.2 ± 1.0	<0.001	3.9 ± 1.1	<0.001	3.0 ± 0.7	<0.001	2.1 ± 1.2	<0.001	1.1 ± 1.0	0.127	12.3 ± 3.2	<0.001
	Professional Associations	2.0 ± 1.0	0.143	3.3 ± 1.3	0.262	2.5 ± 1.1	0.177	1.8 ± 1.4	0.129	1.1 ± 0.9	0.141	10.7 ± 3.9	0.064
	Scientific Journals	2.5 ± 0.7	0.002	4.1 ± 1.0	0.003	2.3 ± 0.7	0.328	2.2 ± 1.1	0.018	1.0 ± 0.8	0.642	12.2 ± 2.7	0.006
	Social Media	1.8 ± 1.2	0.262	3.1 ± 1.6	0.507	2.4 ± 1.1	0.166	1.5 ± 1.2	0.632	0.9 ± 0.9	0.685	9.7 ± 4.7	0.600
	News Portals	1.7 ± 1.3	0.959	3.2 ± 1.5	0.178	2.3 ± 1.1	0.617	1.6 ± 1.3	0.312	1.0 ± 0.9	0.226	9.7 ± 4.4	0.457
Overall Score: μ ± SD (min-max)		1.7 ± 1.1 (0 – 3)		3.0 ± 1.5 (0 – 5)		2.3 ± 1.2 (0 – 4)		1.5 ± 1.2 (0 – 4)		0.9 ± 0.9 (0 – 4)		9.4 ± 4.6 (0 – 20)	

Kruskal-Wallis (H) test and Mann-Whitney (U) test were used with a significance level (*Sig.*) of ≤ 0.05.

Table S4. Predictors of HMPXV vaccine-related Perceptions and Acceptance of Czech Healthcare Workers Responding to HMPXV Survey, September 2022, (n = 341)

Variable	Outcome	Perceived Susceptibility	*Sig.*	Perceived Severity	*Sig.*	Perceived Benefits	*Sig.*	Perceived Barriers	*Sig.*	Cues to Action	*Sig.*	Acceptance	*Sig.*
Gender	Female	6.6 ± 2.4	0.468	9.1 ± 2.2	0.477	9.8 ± 2.1	0.273	6.0 ± 1.7	0.067	9.2 ± 2.6	0.358	2.5 ± 0.9	0.472
	Male	6.5 ± 2.8		9.6 ± 2.5		10.4 ± 2.1		5.3 ± 2.0		10.0 ± 3.2		2.7 ± 1.2	
	Prefer not to say	5.4 ± 2.5		9.6 ± 2.3		10.8 ± 1.6		5.8 ± 1.5		9.6 ± 1.5		2.8 ± 0.4	
Sexual Orientation	Heterosexual	6.6 ± 2.5	0.684	9.1 ± 2.3	0.736	9.8 ± 2.2	0.427	5.9 ± 1.8	0.542	9.3 ± 2.7	0.289	2.5 ± 0.9	0.514
	Homosexual	7.9 ± 3.6		8.5 ± 1.3		10.1 ± 2.6		6.0 ± 1.9		10.8 ± 3.2		3.0 ± 1.2	
	Bisexual	6.6 ± 3.6		10.0 ± 2.3		11.4 ± 2.9		6.8 ± 1.3		10.6 ± 2.9		2.6 ± 1.5	
	Prefer not to say	6.6 ± 2.3		9.3 ± 2.0		10.1 ± 1.4		5.9 ± 1.6		8.7 ± 2.7		2.4 ± 0.8	
Age Group	≤ 47 years-old	6.8 ± 2.6	0.218	9.2 ± 2.3	0.780	9.9 ± 2.2	0.983	6.2 ± 1.9	0.034	9.4 ± 3.0	0.376	2.5 ± 1.0	0.919
	> 47 years-old	6.4 ± 2.4		9.0 ± 2.2		9.8 ± 2.0		5.7 ± 1.5		9.2 ± 2.4		2.5 ± 0.9	
Marital Status	Single	7.0 ± 2.8	0.782	9.4 ± 2.8	0.954	10.2 ± 2.2	0.416	6.0 ± 2.1	0.333	9.9 ± 3.2	0.034	2.8 ± 1.2	0.111
	Married	6.5 ± 2.4		9.0 ± 2.1		9.8 ± 2.2		5.9 ± 1.7		9.4 ± 2.5		2.4 ± 0.9	
	Divorced	6.4 ± 2.6		9.1 ± 1.9		9.5 ± 1.9		6.5 ± 1.6		8.2 ± 2.7		2.3 ± 0.9	
	Widow	6.4 ± 1.6		8.9 ± 1.8		10.3 ± 1.6		6.0 ± 1.2		9.1 ± 1.1		2.3 ± 0.7	
	Prefer not to say	6.8 ± 2.4		9.0 ± 2.6		10.1 ± 1.6		5.8 ± 1.6		8.8 ± 2.5		2.4 ± 0.8	
Having Minors	Yes	6.4 ± 2.4	0.150	8.7 ± 2.2	0.012	9.7 ± 2.2	0.130	5.9 ± 1.8	0.849	8.9 ± 2.7	0.049	2.3 ± 0.9	0.005
	No	6.8 ± 2.5		9.3 ± 2.2		10.0 ± 2.1		6.0 ± 1.7		9.5 ± 2.7		2.6 ± 1.0	
Location	South Moravian Region	6.6 ± 2.4	0.900	9.2 ± 2.2	0.325	9.7 ± 2.0	0.057	6.0 ± 1.6	0.319	9.0 ± 2.7	0.015	2.4 ± 0.9	0.090
	Other Regions	6.6 ± 2.6		9.0 ± 2.3		10.1 ± 2.3		5.9 ± 1.9		9.7 ± 2.6		2.6 ± 1.0	
Profession	Medical	6.6 ± 2.7	0.894	9.3 ± 2.4	0.697	10.1 ± 2.2	0.375	5.5 ± 1.4	0.132	10.3 ± 3.0	0.045	2.8 ± 1.0	0.053
	Allied HCPs	6.6 ± 2.5		9.1 ± 2.2		9.8 ± 2.1		6.0 ± 1.8		9.2 ± 2.6		2.5 ± 0.9	
Providing Care	Yes	7.4 ± 3.3	0.272	9.1 ± 2.4	0.688	9.9 ± 1.8	0.903	6.6 ± 1.8	0.164	10.3 ± 2.5	0.144	2.6 ± 1.0	0.608
	No	6.6 ± 2.4		9.1 ± 2.2		9.9 ± 2.2		5.9 ± 1.7		9.3 ± 2.7		2.5 ± 0.9	
Chronic Illnesses	Yes	6.7 ± 2.6	0.565	9.3 ± 2.1	0.473	10.3 ± 1.9	0.014	6.1 ± 1.8	0.316	9.7 ± 2.5	0.021	2.5 ± 0.9	0.707
	No	6.5 ± 2.4		9.0 ± 2.3		9.6 ± 2.3		5.9 ± 1.7		9.0 ± 2.7		2.5 ± 0.9	
Medical Treatments	Yes	6.6 ± 2.5	0.703	9.3 ± 2.1	0.333	10.2 ± 2.0	0.010	6.0 ± 1.8	0.489	9.6 ± 2.6	0.028	2.5 ± 0.9	0.337
	No	6.7 ± 2.5		8.9 ± 2.3		9.6 ± 2.2		5.9 ± 1.7		9.0 ± 2.7		2.4 ± 1.0	
COVID-19 Vaccination	Yes	6.7 ± 2.5	0.048	9.2 ± 2.2	0.016	10.0 ± 2.1	<0.001	5.9 ± 1.6	0.004	9.6 ± 2.6	<0.001	2.6 ± 0.9	<0.001
	No	5.8 ± 2.0		8.1 ± 2.1		8.6 ± 1.8		6.9 ± 2.4		6.7 ± 2.4		1.8 ± 0.8	
COVID-19 Vaccine Doses	One Dose	5.6 ± 3.6	0.186	8.2 ± 1.9	0.585	7.4 ± 4.3	0.006	6.4 ± 2.3	0.030	7.2 ± 3.9	0.190	2.0 ± 1.0	0.023
	Two Doses	6.4 ± 2.4		9.1 ± 2.2		9.7 ± 2.3		6.4 ± 1.6		9.4 ± 2.4		2.4 ± 0.9	

	Three Doses	6.8 ± 2.5		9.2 ± 2.2		10.0 ± 2.0		5.8 ± 1.6		9.6 ± 2.5		2.6 ± 0.9	
	Four Doses	5.6 ± 2.7		9.6 ± 2.4		11.6 ± 2.2		5.1 ± 2.4		10.7 ± 2.6		3.1 ± 0.9	
Influenza	Yes	6.7 ± 2.6	0.510	9.1 ± 2.4	0.907	10.2 ± 2.3	0.011	5.8 ± 1.8	0.128	9.8 ± 2.8	0.003	2.7 ± 1.0	<0.001
Vaccination	No	6.5 ± 2.4		9.1 ± 2.1		9.7 ± 2.1		6.1 ± 1.7		9.0 ± 2.6		2.4 ± 0.9	
Last 12	Yes	6.8 ± 2.7	0.989	8.9 ± 2.5	0.345	10.5 ± 2.3	0.134	5.7 ± 1.7	0.588	10.0 ± 2.7	0.514	3.0 ± 0.8	0.002
Months	No	6.7 ± 2.5		9.2 ± 2.3		9.9 ± 2.2		5.9 ± 1.8		9.6 ± 2.9		2.4 ± 1.0	
Undergrad	Yes	6.9 ± 2.6	0.529	9.2 ± 1.5	0.607	10.6 ± 2.2	0.160	5.6 ± 1.6	0.428	10.0 ± 2.4	0.223	2.8 ± 0.8	0.093
Curriculum	No	6.6 ± 2.5		9.1 ± 2.3		9.8 ± 2.1		6.0 ± 1.8		9.3 ± 2.7		2.5 ± 0.9	
Utilised Sources	Ministry of Health	6.8 ± 2.2	0.046	9.0 ± 2.1	0.496	10.0 ± 2.1	0.290	5.9 ± 1.8	0.965	9.6 ± 2.7	0.024	2.6 ± 0.9	0.070
	Public Health Institutes	6.7 ± 2.4	0.932	9.0 ± 2.1	0.679	10.2 ± 2.5	0.124	5.8 ± 1.8	0.428	9.7 ± 2.7	0.350	2.7 ± 0.9	0.060
	ECDC	6.5 ± 2.4	0.781	8.8 ± 1.9	0.671	9.9 ± 2.8	0.674	5.3 ± 1.4	0.080	10.1 ± 2.3	0.295	2.9 ± 0.9	0.090
	US CDC	8.8 ± 3.1	0.093	9.2 ± 2.5	0.951	11.8 ± 2.8	0.135	4.8 ± 1.3	0.105	12.0 ± 3.5	0.058	3.6 ± 1.1	0.022
	WHO	6.7 ± 2.4	0.732	9.0 ± 2.3	0.783	10.4 ± 2.3	0.029	5.8 ± 1.9	0.293	9.8 ± 2.4	0.117	2.7 ± 0.9	0.033
	Professional Associations	6.8 ± 2.5	0.855	9.4 ± 2.3	0.296	10.1 ± 2.4	0.192	5.9 ± 1.9	0.420	9.5 ± 2.7	0.960	2.6 ± 1.0	0.497
	Scientific Journals	7.2 ± 2.4	0.276	9.8 ± 2.5	0.293	10.8 ± 1.7	0.052	5.6 ± 1.9	0.492	10.6 ± 2.3	0.050	3.1 ± 0.8	0.004
	Social Media	6.7 ± 2.5	0.638	9.0 ± 2.2	0.414	9.6 ± 1.9	0.097	6.0 ± 1.7	0.557	9.3 ± 2.2	0.696	2.5 ± 0.9	0.695
	News Portals	6.5 ± 2.6	0.385	9.1 ± 2.1	0.553	10.1 ± 2.1	0.152	5.9 ± 1.7	0.737	9.5 ± 2.5	0.391	2.5 ± 1.0	0.368
Overall Score: μ ± SD (min-max)		6.6 ± 2.5 (3 – 15)		9.1 ± 2.2 (3 – 15)		9.9 ± 2.1 (3 – 15)		6.0 ± 1.7 (2 – 10)		9.3 ± 2.7 (3 – 15)		2.5 ± 0.9 (1 – 5)	

Kruskal-Wallis (H) test and Mann-Whitney (U) test were used with a significance level (*Sig.*) of ≤ 0.05.
